# Fast Pyrolysis of Tropical Biomass Species and Influence of Water Pretreatment on Product Distributions

**DOI:** 10.1371/journal.pone.0151368

**Published:** 2016-03-15

**Authors:** Trevor James Morgan, Scott Q. Turn, Ning Sun, Anthe George

**Affiliations:** 1 Hawaii Natural Energy Institute, University of Hawaii at Manoa, Honolulu, Hawaii, 96822, United States of America; 2 Lawrence Berkeley National Laboratory, Berkeley, California, 94720, United States of America; 3 Combustion Research Facility, Sandia National Laboratories, Livermore, California, 94550, United States of America; National University of Ireland - Galway, IRELAND

## Abstract

The fast pyrolysis behaviour of pretreated banagrass was examined at four temperatures (between 400 and 600 C) and four residence times (between ~1.2 and 12 s). The pretreatment used water washing/leaching to reduce the inorganic content of the banagrass. Yields of bio-oil, permanent gases and char were determined at each reaction condition and compared to previously published results from untreated banagrass. Comparing the bio-oil yields from the untreated and pretreated banagrass shows that the yields were greater from the pretreated banagrass by 4 to 11 wt% (absolute) at all reaction conditions. The effect of pretreatment (i.e. reducing the amount of ash, and alkali and alkali earth metals) on pyrolysis products is: 1) to increase the dry bio-oil yield, 2) to decrease the amount of undetected material, 3) to produce a slight increase in CO yield or no change, 4) to slightly decrease CO_2_ yield or no change, and 5) to produce a more stable bio-oil (less aging). Char yield and total gas yield were unaffected by feedstock pretreatment. Four other tropical biomass species were also pyrolyzed under one condition (450°C and 1.4 s residence time) for comparison to the banagrass results. The samples include two hardwoods: leucaena and eucalyptus, and two grasses: sugarcane bagasse and energy-cane. A sample of pretreated energy-cane was also pyrolyzed. Of the materials tested, the best feedstocks for fast pyrolysis were sugarcane bagasse, pretreated energy cane and eucalyptus based on the yields of 'dry bio-oil', CO and CO_2_. On the same basis, the least productive feedstocks are untreated banagrass followed by pretreated banagrass and leucaena.

## Introduction

Hawaii is the most fossil fuel dependent state in the United States. The 'Hawaii Clean Energy Initiative' aims to address this with a goal of producing 70% of the state's energy from clean energy sources by 2030 [1]. Biomass fast pyrolysis is one of the potential pathways under investigation. Fast pyrolysis of woody biomass produces significant amounts of bio-oil, up to ~75 wt% of the dry-ash-free (daf) feedstock [2–7]. However, woody biomass species are typically less productive [8], i.e. lower annual dry matter yield, than herbaceous species. For this reason and in order to utilize biomass produced across the available landscapes, there is interest in the use of grasses and agricultural residues as fast pyrolysis feedstock's [2, 9–12].

Fast pyrolysis of agricultural residues, such as straws, tends to produce less bio-oil than woods or grasses (typically <50 wt% bio-oil from straws) [2, 9]. Sugarcane bagasse tends to produce significantly more bio-oil than straws. Although, the bio-oil yield from sugarcane bagasse can be highly variable depending on the growing conditions and the way cane is harvested, i.e. soil can be incorporated with the cane. Bio-oil yields from sugarcane bagasse typically range from ~50 to 70 wt% [13–17]. Grasses such as switchgrass and miscanthus (energy crops) also produce fairly high bio-oil yields, falling within the lower end of the range seen for woody biomass (~50–60 wt% bio-oil) [9–12].

*Pennisetum* is a genus of tropical and warm temperate grasses. Banagrass used in the present study is a cross between napier grass *(Pennisetum purpureum*) and pearl millet (*Pennisetum glaucum*) capable of producing 40 to 65 Mg fiber ha^-1^ yr^-1^ with nonirrigated and irrigated conditions representing the lower and higher end of this range, respectively [18–20]. In comparison, typical yields from switchgrass are 5 to 15 Mg fiber ha^-1^ yr^-1^ which can reach up to ~38 Mg fiber ha^-1^ yr^-1^ with longer growing seasons and ample water resources [21]. The higher heating value (HHV) of banagrass ranges from 17 to 18.5 MJ/kg (present study and reference [22]) which makes it an interesting prospect as a nonfood crop suitable for energy applications.

Banagrass has a high ash content (~4–15 wt% dry basis, typically ~9 wt%) and high content of alkali and alkali earth metals (AAEM) compared to most other types of biomass [20, 22, 23]. For example, woods typically contain low amounts of ash, <2–3 wt% of the dry feedstock [23]. Switchgrass and miscanthus can contain between ~2–6 wt% ash [9, 10, 12], elephant grass ~5–7 wt% ash [24, 25], and napier grass ~3–9 wt% ash [26, 27]. A review of the composition of more than 80 biomass species has been reported [23]. It has recently been demonstrated that modern X-ray fluorescence instruments can provide data that is comparable to inductively coupled plasma methods for the inorganic composition (major and minor elements) of raw biomass, i.e. without the need to ash the sample [28, 29].

Ash chemistry plays an important role during biomass pyrolysis. Inorganic elements, and AAEM in particular, are responsible for catalyzing cracking reactions of biomass pyrolysis vapors which reduce bio-oil yields [2, 6, 9, 30]. Shafidazeh et al. [31] were possibly the first researchers to show that a mild acid washing of wood powders before pyrolysis increased the yield of laevoglucosan. It was suggested that the improved bio-oil yield after acid washing was related to a reduction in potassium and magnesium which reduced cracking reactions.

Oasmaa et al. [2] found that the amount of ash, and in particular, the amount of alkali metals (Na + K) correlated with the amount of bio-oil that was produced via fast pyrolysis. Lower bio-oil yields were recovered for biomass species that contain more ash. Potassium and sodium are thought to be catalytic leading to increased cracking of bio-oil compounds into volatiles, gases and water. Biomass species with high ash contents also tend to produce a less stable bio-oil (more aging) [2, 12].

Mourant et al. [5] looked at the influence of AAEM on the fast pyrolysis of mallee wood (*Eucalpytus loxophleba*), ash content < 1 wt%. Mallee wood was washed with water or acid to remove AAEM. The results showed that reduction of AAEM did not significantly alter bio-oil or char yields, which is possibly due to the low amount of ash and AAEM originally present in mallee wood. Reduction of the AAEM species increased viscosity, and increased yields of sugars and lignin derived oligomers. Washing also decreased the amount of pyrolysis water and light organic compounds in the bio-oil. Acid-washing was found to have a greater affect than water washing, with more Ca removed via acid washing.

Fahmi et al. [11] investigated the role of alkali metals and lignin on the pyrolysis of grasses (*Lolium* and *Festuca* varieties). The grasses where genetically mutated to contained differing amounts of lignin, between 2 and 6%. Samples were also washed with water to reduce their ash content. The main findings were: 1) K and Na induced a strong catalytic effect on bio-oil cracking activity; 2) as the lignin content increased, the metals content decreased; 3) as metals content increase char yields increase; and 4) less metals result in increased laevoglucosan yield and less hydroxyacetaldehyde. These findings support the mechanism suggested by Liden et al. [32], where AAEM promote an ionic route that favors ring-scission and formation of hydroxyacetaldehyde. However, Fahmi's study [11] was carried out using a thermogravimetric analyzer (TGA) and a pyrolysis-GCMS instrument which may give unreliable results compared to fast pyrolysis in a fluidized bed.

A follow up study by Famhi et al. [12] used a fluidized bed reactor to better understand their findings from TGA and PY-GCMS. The main findings were: 1) The role of metals is more dominant than the lignin concentration. 2) The high molecular mass species present in pyrolysis oil are mainly from the lignin. 3) As the amount of metals increases, the light fraction increases (greater cracking) and less pyrolysis water is formed; 4) as the concentration of metals decrease, more gas is formed. In general, for biomass with high ash content, the quantity, quality and stability of the bio-oil improves after washing. The effect was less significant for switchgrass which contains low amounts of AAEM. Water washing of switchgrass resulted in a marginally lower viscosity (or no change), whereas for *Festuca arundinace* [12] the viscosity increased matching the behaviour of mallee wood [5].

Considering the studies mentioned above, some general trends can be drawn where lowering the concentration of AAEM results in: 1) more bio-oil, 2) less volatiles, 3) less pyrolysis water, 4) less gas, 5) more char, 5) increased bio-oil viscosity, 6) a more stable bio-oil (less aging). However, there is some inconsistency in the findings from different researchers. Oasmaa [2] reported that decreasing AAEM results in less gas, whereas Fahmi [12] reported more gas, and Mourant [5] saw no effect. Oasmaa [2] and Mourant [5] also reported that decreasing amounts of AAEM results in less pyrolysis water but Fahmi [12] found the opposite. Fahmi [11] also reported that decreasing the amount of AAEM produces more char whereas the other groups did not report that finding. The reason for these discrepancies are unclear but may be related to differences between the biomass varieties being examined, and the difficultly of simultaneously obtaining accurate data (amounts and composition) for all of the products from pyrolysis. Differences in the reactor systems used (despite all being fluidized bed reactors), as well as differences in operating conditions would also probably affect the variability in the results discussed above.

The amount of gas formed during biomass fast pyrolysis is also thought to be related to the hemi-cellulose contents of the feedstock, where higher amounts of hemi-cellulose lead to more acids and gases being formed [2]. More pyrolysis water is typically formed from hardwoods than softwoods under equivalent conditions, which is explained in terms of the hemi-cellulose in hardwoods containing more acetylated groups than softwoods which dehydrate during thermal decomposition producing water and furfural [4].

There are few reports on the pyrolysis of banagrass [20] or of *Pennisetum purpureum* species in general (elephant or napier grass). Only one fast pyrolysis study was found for elephant grass [24]. However, that study focused on charcoal production; bio-oil and permanent gas results were not reported. A couple of slow pyrolysis studies of napier and elephant grasses using fixed bed reactors have also been reported [25–27, 33].

Braga et al. [25] reported on the slow pyrolysis of elephant grass (*Pennisetum purpureum Schum*) after pretreatment via hot water or acid washing. Washing reduced the ash content from 6.9 wt% (wet basis) to 2.5 wt%, although the composition of the ash was not reported. Washing increased the volatiles yield from 77 wt% (wet basis) to 85 wt%. The washing also appeared to reduce the activation energy required to decompose elephant grass. However, the study was carried out using a thermogravimetric analyzer (TGA) and the maximum heating rate was 30°C/min which makes their findings difficult to interpret. The problems associated with using TGA's for these types of study have been discussed elsewhere [6].

In our previous work, the fast pyrolysis of banagrass was examined in a fluidized bed reactor as a function of temperature (400 to 600°C) and volatiles residence time (~1.2 to 12 s) [34]. In the present study, the effect of a mild pretreatment on banagrass pyrolysis was examined using the same reactor system and reaction conditions (i.e. 400–600°C and ~1.2 to 12 s residence time). The pretreatment used water washing/leaching to reduce the inorganic content of the banagrass. Four other tropical biomass species were also pyrolyzed under one condition (450°C and 1.4 s residence time) for comparison to the banagrass results. The samples include two hardwoods: leucaena and eucalyptus, and two grasses: sugarcane bagasse and energy-cane. A sample of pretreated energy-cane was also pyrolyzed. The samples used in this study were grown in the State of Hawaii.

## Experimental

### Sample preparation

Banagrass *(Pennisetum purpureum* x *Pennisetum glaucum)* was harvested from the Waimanalo Experiment Station of the University of Hawaii. The above ground material was processed into nominal 50 mm pieces (stalk and leaves) using a shredder (Vincent Corp., Tampa, FL) and dried to equilibrium moisture content (~10% moisture) using an ambient air drying bed. Energy cane *(Saccharum spontaneum)* was harvested from experimental plots at the Hawaiian Commercial & Sugar Co. (HC&S) at Puunene on Maui. The above ground portion was processed in the same manner as banagrass.

Eucalyptus (*Eucalyptus grandis*) was harvested from a ~10 year old commercial forest north of Hilo on the island of Hawai'i, shipped to Oahu by interisland barge, chipped (Model 10, Morbark, Winn, MI) and then dried in a forced air ambient temperature fixed bed drier. The eucalyptus was acquired from private land with the permission of the timber management company, no specific permissions were required for these activities. Leucaena (*Leucaena leucocephala*) was harvested from University of Hawaii experimental plantings in Waimanalo, Hawaii, and processed as described for eucalyptus. Sugarcane bagasse (*Saccharum officinarum L*.) was obtained from the HC&S sugar factory (Puunene, Maui) and dried in a fixed bed ambient air dryer.

*Pretreatment*: The pretreatment processing method has been described elsewhere [35]. Briefly, liquids were removed from samples of shredded banagrass and energy cane using a screw press to generate a solid fuel sample (S1) and initial plant juice (L1). The S1 samples were loaded in a basket with walls made from 100 mesh screen and immersed in a barrel filled with enough tap water to submerge all of the material. Room temperature tap water (~25°C) was used to leach the solid (S1) at a water to solid mass ratio of ~6.0. Leaching and draining free leachate (L2) from the material were completed in ~5 minutes. Once drained of free leachate, the leached solid material (S2) was subjected to a second dewatering treatment with a screw press to produce the final solid fuel (S3) and a liquid stream (L3). In the present study, the 'pretreated' sample refers solely to the final solid fuel which is labeled S3. Fast pyrolysis of the untreated banagrass has previously been examined [34]. A sample of untreated (S0) energy cane was also pyrolyzed during the present study.

A representative sub sample of ~200 g from each biomass was ground to particle sizes that passed through a 200 μm screen for the pyrolysis experiments. The proximate analysis (ASTM D 3172), ultimate analysis (ASTM D 3176), ash composition (ASTM D3682, 600°C) and heating values of the samples were determined at an accredited laboratory (Hazen Research Inc., Golden, CO). Total carbohydrate and lignin content (compositional analysis) of the samples was measured in triplicate using the National Renewable Energy Laboratory procedure TP-510-42618 and instrumentation described elsewhere [36, 37]. The relative standard deviation of the results from compositional analysis are < 10%.

### Fluidized bed reactor

**Fig 1:**
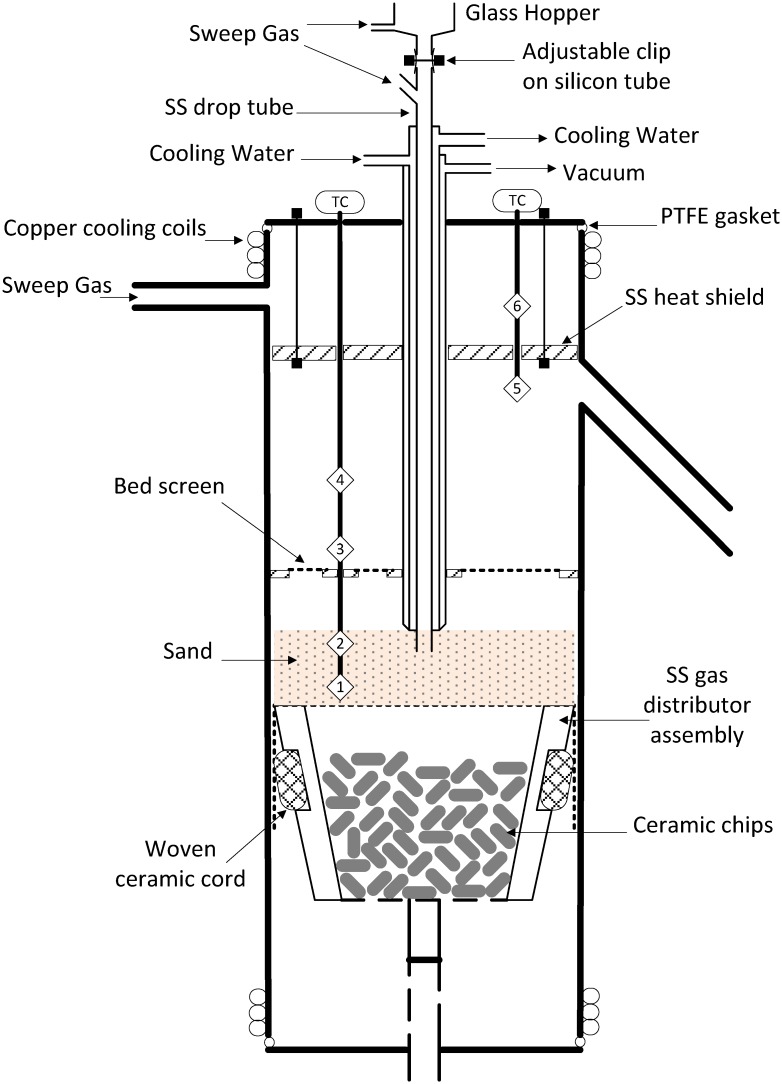
Schematic diagram of the variable-freeboard pyrolysis reactor. Numbers 1 through 6 show the locations of the thermocouple measurements in the multi-point temperature probes (SS—stainless steel).

The fluidized bed reactor is shown in Fig 1; a detailed description has previously been reported [34]. Briefly, the reactor includes an inert sand fluidized bed, a moveable bed support plate, a mesh screen to retain char and fines in the bed, and a side-arm for removing gas phase volatiles from the free-board. The reactor design is based on those in literature [38, 39]. Approximately, 500 g of fresh sand was used as bed material for each experiment (Acros Organics, USA, acid washed sand, average particle size 205 μm, code number: 37094–0000). The elemental composition of the sand is SiO_2_: 99.8%, Fe_2_O_3_: 0.009%, Al_2_O_3_: 0.040%, TiO_2_: 0.016%, K_2_O: 0.006%, CaO: 0.005%, Cr_2_O_3_: 0.00005% (analysis provided by the supplier).

Fluidization velocities of 2.75 to 3.75 times the minimum fluidization velocity were used. Four bed positions were used to produce different volatiles residence times without altering the fluidizing conditions; corresponding residence times (RT) at each bed position (BP) and temperature are given in Table 1. Residence times are based on the dimensions of the freeboard and do not include the time taken for the vapors to pass through the side-arm to the bio-oil traps. Including the side-arm volume increases the RT by ~0.5 seconds.

**Table 1:** Volatiles residence times (s) at the flow rates (LPM, STP) to achieve minimum fluidization velocity, for the four different bed positions and four temperatures used in this study. The times are derived from the volume of the freeboard alone, excluding the side-arm where the volatiles pass to the bio-oil traps.

Temperature	Flow rate	Bed Position			
°C	SLPM	BP-1	BP-2	BP-3	BP-4
		Seconds			
400	~2.7	12.2	8.3	4.6	1.5
450	~2.5	11.3	7.7	4.2	1.4
500	~2.3	10.6	7.2	4.0	1.3
600	~2.0	9.4	6.4	3.5	1.2

A stainless steel water cooled sample drop-tube with an additional, outer, vacuum jacket is used to transfer the feedstock from a glass hopper into the bed. The heating rate of the feedstock as it enters the bed is estimated to be ~400°C/s. The temperature distribution across the bed and freeboard at each reaction condition (bed position and nominal reaction temperature) are presented in Tables A to D in S1 File.

A wire-mesh screen attached to the drop tube, denoted as the 'bed screen,' is located ~5 cm above the stationary bed to prevent losses of char from the bed—(see Fig 1). An additional wire-mesh screen was placed in the flange gasket that connects the exit side-arm to the first bio-oil trap. The side-arm is heat traced from the exit of the furnace to the flange to maintain a gas temperature of 340–360°C. The flange connecting the side arm and the traps is cooled with dry-ice to quench the exiting vapors and collect them in two, liquid-nitrogen cooled, stainless steel traps in series. A slip-stream of the permanent gases exiting the second bio-oil trap passes through a train of on-line gas analyzers.

### Experimental procedure

A detailed account has been previously reported [34]. Briefly, the feedstock was fed into the bed from a gravity-flow hopper connected to the top of the drop-tube. Approximately 7.5 g of feedstock was used for each experiment and took 1.5 to 2.5 minutes to feed into the reactor. In all cases, heating and all gas flows were stopped 15 minutes after fuel feeding was initiated. Immediately after stopping the experiment, the oil traps were removed from the reactor at the side-arm flange and the bio-oil recovered.

Bio-oil was recovered from the traps by washing with a mixture of 80 vol. % acetone and 20 vol. % methanol (HPLC grade, Fisher Chemicals). Liquids from the soxhlet extraction of the thimble from the first oil trap and the rinse from the first oil trap were combined. The rinse from the second oil trap was analyzed separately. Bio-oil solutions were filtered after recovery (Whatman, UK, part number: 1004090).

Samples of the bio-oil solutions (trap-1 and trap-2) were analyzed separately by GCMS. The two bio-oil solutions were stored overnight at -20°C. A rotary evaporator operating at 55°C with a nitrogen purge and a maximum vacuum of ~25" Hg was used to remove the solvent. Three sub-samples from each bio-oil solution were dried and the average of these determinations is defined here as the 'dry' bio-oil yield. A sample of the dry bio-oil was dissolved in fresh solvent and analyzed by GCMS. Comparing this dry bio-oil analysis with the analysis of bio-oil solutions before they were dried provides an estimate of the ‘volatile bio-oil’ fraction removed with the solvent during rotary evaporation. The repeatability and bias of the 'volatile bio-oil' yield is discussed in the GCMS experimental section. Rotary evaporation resulted in water being removed from the bio-oil samples, therefore determination of pyrolysis water was not attempted.

The reported char yields included char recovered from the bed, the soxhlet thimble and by filtering the bio-oil solutions. Char samples were ashed in a muffle furnace at 600°C, accordingly, char yields were corrected to a dry ash free (daf) basis. Char yields are reported for the organic fraction (Char_Org_) excluding ash, i.e. on a daf basis relative to the daf feedstock. Char yields are also reported inclusive of ash (Char_Org+Inorg_) on a dry basis relative to the dry feedstock.

### Uncertainty, repeatability and bias

The results presented herein are based on a single pyrolysis experiment at each condition. The repeatability and bias of the results obtained from the pyrolysis reactor were examined in a previous study where three to four repeat experiments were performed with cellulose and banagrass [34].

Repeatability (standard deviation) of the dry bio-oil yield is ≤±2 wt% (absolute) of the daf feedstock [34]. There may also be some bias in results as discussed in our previous publication [34], where the bio-oil yield may be underestimated by ~10 wt% (absolute) at 400°C and ~5 wt% at 600°C. The repeatability of the char yield determinations is < ±1.5 wt% (absolute). The bias in the char yield is estimated to be < ±2.0 wt% (absolute). The uncertainty associated with the permanent gas yields are discussed below.

### Permanent gas analysis (CO, CO_2_, CH_4_ and H_2_)

Permanent gases were analyzed using two online gas analyzers, 1) a Uras 10E three channel non-dispersive infrared analyzer for CO, CO_2_ and CH_4_, and 2) a Caldos 5G continuous flow thermal conductivity detector for H_2_. The analyzers were supplied by Applied Automation/Hartmann & Braun, Bartlesville, USA. The detectors were calibrated using certified zero and span gases.

Values reported for permanent gases can only be considered as 'indicative' due to cross interference between gas species and the transient nature of the gases emitted from the batch pyrolyzed sample. The bias in the permanent gas yield determinations is estimated to be ± 10% (relative). The repeatability of the permanent gas yields was surprisingly good, see results section. A more thorough account of the uncertainty associated with these measurements has been described elsewhere [34]. The term 'permanent gases' refers solely to CO, CO_2_, CH_4_ and H_2_ and not to other gases formed during pyrolysis.

### GCMS

A Bruker SCION GCMS with a BPx-1701 column (Restek corp., USA. 60 m x 0.25 mm, x 0.10 μm) was used to analyze the bio-oils. The analysis used an injector temperature of 280°C. The column temperature was held at 40°C for 4 minutes, ramped to 280°C at 3°C/min and then held at 280 for 20 minutes. The conditions are based on those reported by Mohan et al. [40]. A certified standard (Restek corp., USA) was used to produce a calibration curve for 17 individual compounds: cyclohexane (CAS 110-82-7), furfural (CAS 98-01-1), 3-methyl-2-cyclopenten-1-one (CAS 2758-18-1), phenol (CAS 108-95-2), 4-methylphenol (p-cresol, CAS 106-44-5), 2-methylphenol (o-cresol, CAS 95-48-7), 3-methylphenol (m-cresol, CAS 108-39-4), 2-methoxyphenol (guaiacol, CAS 90-05-1), 2,4-dimethylphenol (CAS 105-67-9), 4-ethyl phenol (CAS 123-07-9), 2-methoxy-4-methyl phenol (creosol, CAS 93-51-6), indole (CAS 120-72-9), 2-methoxy-4-(prop-1-en-1-yl)phenol (isoeugenol, CAS 97-54-1), 2,6-dimethoxy phenol (CAS 91-10-1), 2,2-dimethoxy propane (CAS 77-76-9), benzene (CAS 71-43-2) and naphthalene (CAS 91-20-3). A six point calibration covered the concentration range of ~5 to ~150 μg/mL. Dodecane (CAS 112-40-3) was used as an internal standard.

Each bio-oil solution was analyzed in triplicate so that the relative standard deviation (RSD) could be determined, with regular blank runs (solvent only) performed to avoid carryover of species from one sample to the next. The RSD derived from the calibration and sample data was ≤± 5%. The lower limit of quantification (LLQ) for each compound in the calibration is presented in Tables A-E in S2 File. The LLQ is defined as the point where the calibration became unreliable, i.e. where the RSD exceeded ± 20%.

Note: The compounds removed from the bio-oil samples during rotary evaporation are referred to as 'volatile bio-oil' in the tables. In almost all cases the 'volatile bio-oil' yields are below the LLQ for the calibrated compounds (17 compounds). The reason for these low concentrations is mainly due to the amount of solvent required to recover the bio-oil from the traps (~1 L). If each calibrated compound was present in the sample at the LLQ it would introduce a total bias of ~2.0 wt% to the volatile bio-oil yield relative to the feedstock (daf). However, this '~2 wt%' bias is probably a gross underestimate due to the low concentration of the solutions (~3 mg/mL) which is further exacerbated as less than half the peaks observed in the GC chromatograms are calibrated. The concentration of the bio-oil solutions would have to be increased by an order of magnitude to obtain more reliable GCMS data.

### Elemental analysis of the bio-oils and char (CHN)

The carbon, hydrogen and nitrogen contents of the 'dry bio-oil' and char samples were determined by combustion analysis (Exeter Analytical Model CE 440 elemental analyzer). Using 3 to 6 mg subsamples, a minimum of five (typically eight) analyses were performed on each dry bio-oil and char sample. The standard deviations associated with these analyses are reported in the results section. The bias of the instrument, based on analysis of known samples, is on the order of 1.0% relative [41].

## Results and Discussion

### Results—Feedstock properties

**Fig 2:**
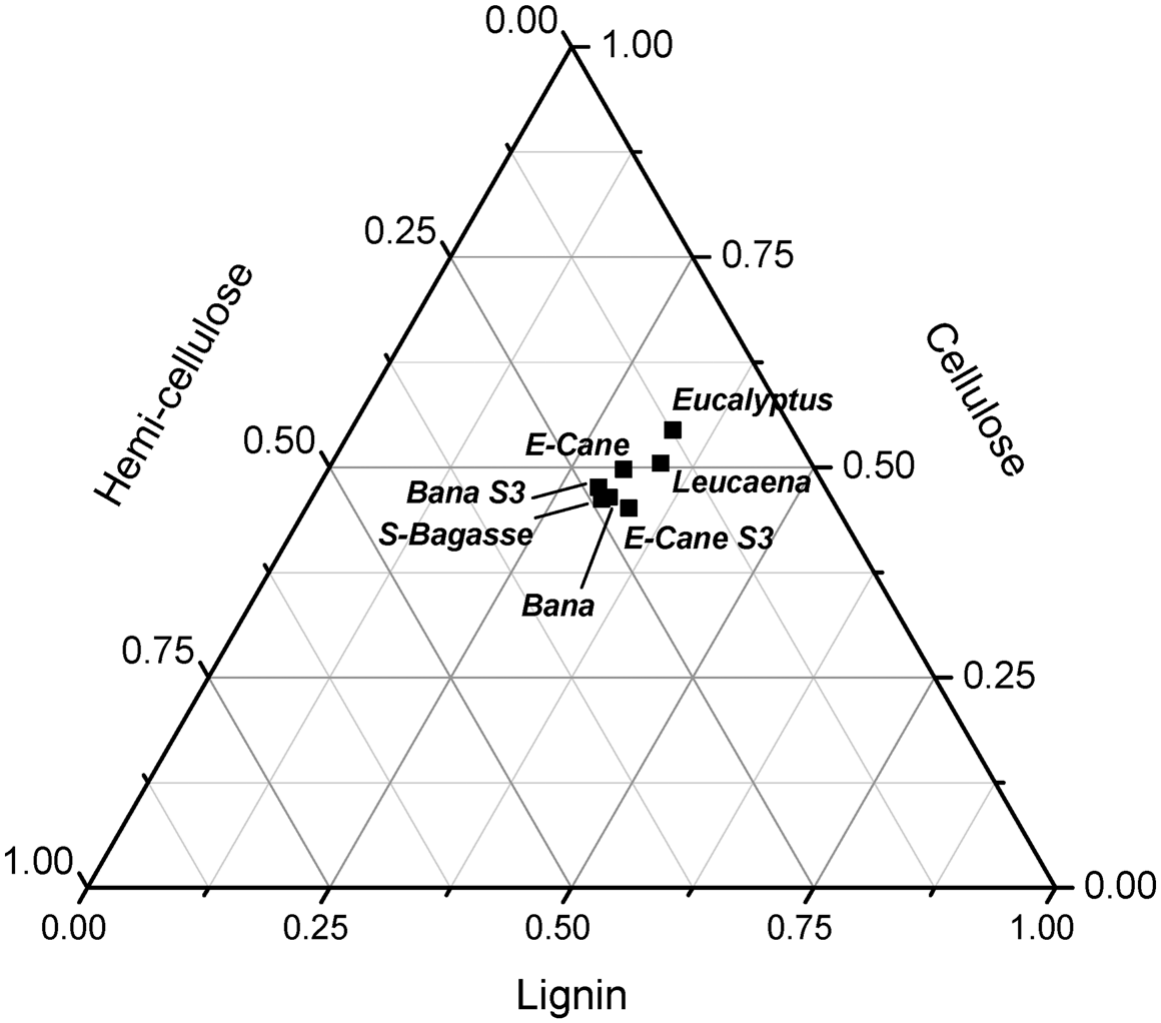
Ternary plot of the compositional analysis of biomass samples examined in this study—using the approach proposed by Vassilev et al. [23]. The data from Table 2 was normalized to 100% before plotting the points.

**Fig 3:**
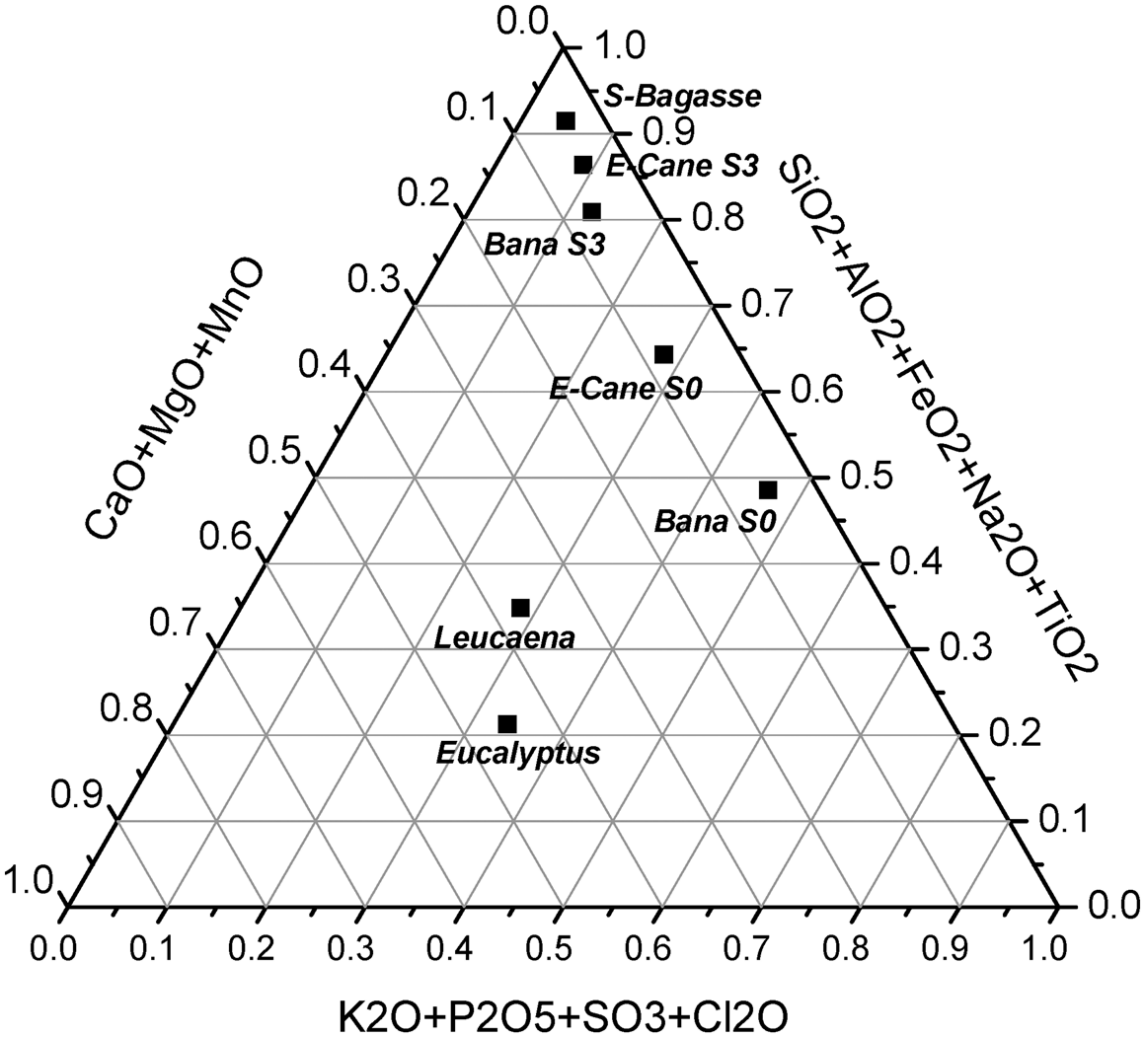
Ternary plot of the ash forming elements in the biomass samples—using the approach proposed by Vassilev et al. [23]. The data from Table A in S3 File was normalized to 100% before plotting the points (wt% of the ash), and no data was available for MnO.

The fuel properties (proximate, ultimate and compositional analysis, and heating values) of the biomass feedstocks used in this study are presented in Table 2. The label 'S3' refers to pretreated samples which have undergone water washing. Fig 2 presents the results from compositional analysis in a ternary plot. The elemental analysis of the ashes from the various feedstock's are displayed in a ternary plot as wt% of the ash in Fig 3 and in Table 3 as wt% of the dry feedstock. The data plotted in Fig 3 is tabulated in Table A in S3 File.

**Table 2:** Fuel properties of leucaena, eucalyptus, sugarcane bagasse, energy cane, pretreated energy cane (S3), banagrass and pretreated banagrass (S3).

	Leucaena	Eucalyptus	S-Bagasse	E-Cane	E-Cane S3	Banagrass	Banagrass S3
Moisture^#^ wt%	6.2	6.1	5.5	6.7	5.6	3.0	0.5^α^
Proximate analysis (wt% dry basis)^β^							
Ash	1.5	0.7	7.6	6.6	3.2	8.5	5.1
Volatiles	83.2	86.3	82.4	78.7	86.4	83.3	84.6
Fixed C	15.3	13.0	10.0	14.7	10.4	8.3	10.4
SUM	100.0	100.0	100.0	100.0	100.0	100.1	100.0
Heating values (MJ/kg dry basis)							
HHV	18.9	18.4	18.0	17.1	18.6	16.8	18.5
LHV	17.6	17.1	16.8	15.9	17.4	15.7	17.2
Ultimate analysis (wt% dry-ash-free basis)^β^							
Carbon	49.8	50.3	51.7	50.4	53.0	51.1	52.3
Hydrogen	6.1	6.0	6.0	5.9	5.9	5.7	6.0
Nitrogen	0.3	0.1	0.5	0.4	0.3	0.5	0.2
Sulfur	0.02	0.05	0.04	0.32	0.05	0.11	0.03
Oxygen*	43.7	43.5	41.9	42.6	40.7	41.2	41.4
Chlorine	0.09	0.06	0.02	0.3	0.01	1.3	0.03
SUM	100.0	100.0	100.0	100.0	100.0	100.0	100.0
Compositional analysis (wt% dry-ash-free basis)^ε^							
Lignin	28.0	26.7	25.5	22.7	26.9	23.5	22.5
Cellulose	41.5	43.7	39.2	37.0	36.3	35.5	36.9
Hemi-cellulose	12.8	9.9	20.2	14.7	17.3	17.5	18.1
SUM	82.2	80.3	84.9	74.4	80.5	76.5	77.5

# Moisture content of the biomass samples after grinding to <200 μm particle size

*Oxygen by difference

^α^ Banagrass S3, this sample was oven dried to aid grinding.

^β^ Standard deviation is estimated to be < 0.5 wt% of the absolute values

^ε^ Relative standard deviation is < 10%.

**Table 3:** Elemental analysis of the ash from leucaena, eucalyptus, sugarcane bagasse, energy cane, energy cane S3, banagrass and banagrass S3, the ash was calcined at 600°C prior to analysis. Presented as wt% of the dry feedstock.

Sample	Element	SiO_2_	Al_2_O_3_	TiO_2_	Fe_2_O_3_	CaO	MgO	Na_2_O	K_2_O	P_2_O_5_	SO_3_	Cl	CO_2_
Leucaena	wt%	0.3	0.1	0.002	0.11	0.4	0.1	0.03	0.3	0.1	0.01	0.10	0.1
Eucalyptus	wt%	0.02	0.02	0.001	0.04	0.2	0.04	0.04	0.1	0.1	0.01	0.01	0.1
S-bagasse	wt%	3.0	1.6	0.30	1.5	0.2	0.1	0.1	0.2	0.1	0.1	0.001	0.02
E-Cane	wt%	4.1	0.1	0.001	0.03	0.4	0.1	0.1	0.9	0.2	0.5	0.20	0.02
E-Cane S3	wt%	2.1	0.04	0.003	0.06	0.1	0.03	0.02	0.1	0.05	0.05	0.001	0.01
Banagrass	wt%	4.0	0.1	0.001	0.04	0.2	0.2	0.04	2.3	0.5	0.1	1.00	0.1
Banagrass S3	wt%	3.1	0.1	0.001	0.09	0.2	0.1	0.03	0.3	0.1	0.04	0.02	0.1

The results in Table 2 show that the woods have greater HHV than the grasses. Pretreatment of banagrass and energy cane increased their heating values to similar levels as the woods. The main effect of pretreatment was to decrease the amount of ash and, and as a result increase the percentage of volatiles. For energy cane, pretreatment reduced the amount of fixed carbon, whereas for banagrass fixed carbon increased (the reason for this is unclear). Pretreatment also reduced the amounts of chlorine, sulfur and possibly nitrogen, and concomitantly increase the percentage of carbon.

The compositional analysis (Table 2, Fig 2) shows that pretreatment results in a slight increase (or no change) in the amount of lignin, cellulose and hemi-cellulose which is more significant for energy cane than banagrass. This is thought to be due to the pretreatment removing some of the extractives from the samples. All the samples fall into the CLH type classification; i.e. based on the order of decreasing content of the components [23]. A review of > 80 biomass varieties has shown that straws, grasses and sugarcane bagasse tend to be CHL type; although elephant grass is HLC type. Woods are typically CLH type [23]. In comparison to literature [23], our samples contain relatively low amounts of hemi-cellulose which matches previous analyses of these samples [8]. The hardwoods have the highest cellulose content with energy cane and banagrass having the lowest. The hardwoods and pretreated energy cane have the highest lignin contents. For hemi-cellulose, sugarcane bagasse, pretreated energy cane and untreated or pretreated banagrass have the highest contents. Overall, the differences between the samples are relatively small as can be seen in Fig 2.

**Fig 4:**
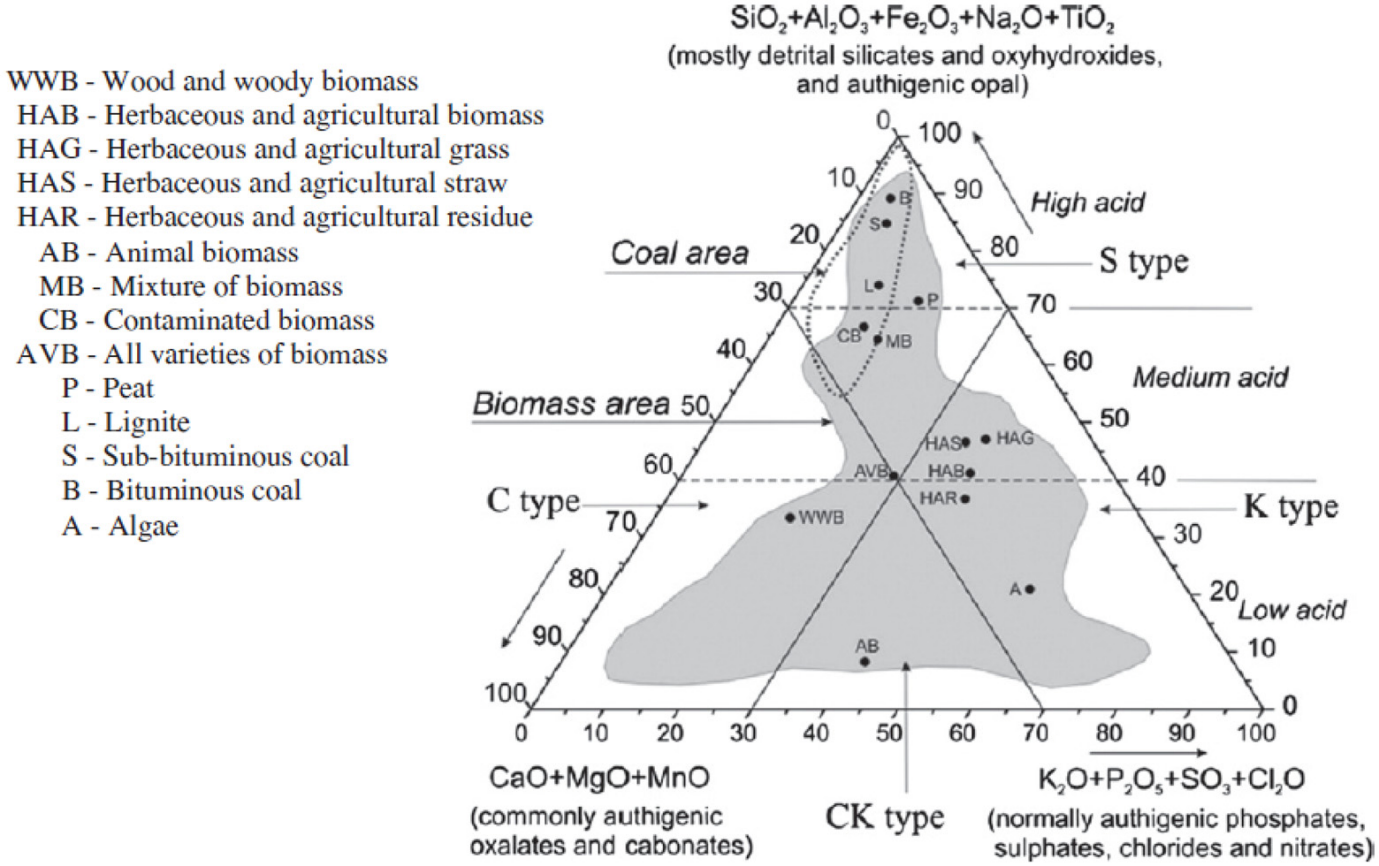
Ternary plot showing position areas of 86 biomass samples and 38 solid fossil fuels in the chemical classification system of the inorganic matter in biomass, wt.% [23]. [Reproduced with permission from Vassilev et al., Fuel 94 (2012) 1–33].

The ternary plot of ash forming elements (Fig 3) shows that eucalyptus and leucaena are located in a region typical for woody biomass species. Refer to reference [23] for the locations and classification of > 80 biomass varieties based on plots of their ash composition, Fig 4 shows the positions of the main biomass and coal regions (reproduced from reference [23]). The untreated banagrass and energy-cane are in the region typical for 'herbaceous and agricultural grasses' [23]. Whereas, after pretreatment the banagrass and energy-cane (S3) ashes are more similar in composition with rice husks and coals—mostly due to the high proportion of silica. The ash from sugar-cane bagasse also falls in the coal region close to bituminous coals which is the result of high concentrations of iron and alumina due to soil incorporation with the sugarcane during harvest. Biomass that is located in the coal region of the ternary plot typically have high melting points and are unlikely to cause deposition problems during combustion—although they are abrasive. For banagrass, its high potassium content is likely to cause deposition problems during combustion even after pretreatment. Further details of the conclusions that can be drawn from the ternary plot can be found elsewhere [23].

Table 3 shows that Si is the dominant inorganic element in the grasses accounting for ~4 wt% of banagrass and energy cane (dry basis) and 3 wt% for sugarcane bagasse which is similar to the amount in the pretreated banagrass and energy cane. Potassium is the next most dominant element with 2.3 wt% in banagrass and 0.9 wt% for energy cane, which are reduced to 0.3 and 0.1 wt% respectively, after pretreatment which is similar to the amount of K in sugarcane bagasse. Sugarcane bagasse contains 1.6 wt% Al, whereas all the other samples contain ≤ 0.1 wt% Al. Pretreatment of banagrass and energy cane reduces the amount of Si, K, P, Na, Ca, Mg, Cl and S.

### Results—Pretreated banagrass pyrolysis, bio-oil, char and gas yields

**Fig 5:**
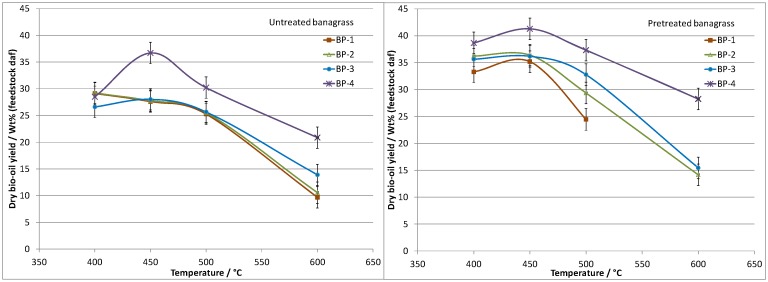
Pyrolysis bio-oil yields (dry bio-oil, daf feedstock) from banagrass as a function of temperature and residence time (bed position, BP). Left side shows untreated banagrass and right side pretreated banagrass.

**Fig 6:**
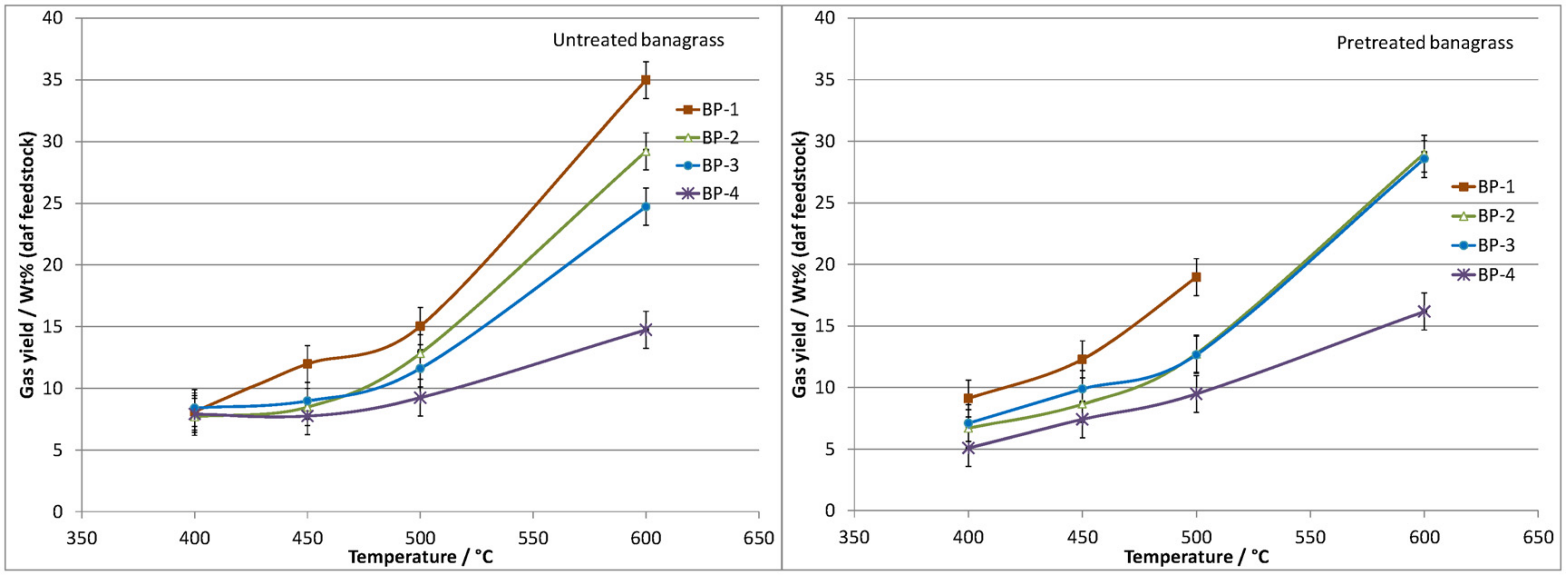
Permanent gas yields (daf feedstock) from banagrass as a function of temperature and residence time (bed position, BP). Left side shows untreated banagrass and right side pretreated banagrass.

The bio-oil and permanent gas yields obtained from the pyrolysis of banagrass before and after pretreatment are presented in Figs 5 and 6, respectively. A summary of the char yields from the pretreated banagrass are given in Table 4. Full data tables for the pretreated banagrass bio-oil, char and gas yields are provided in Tables A to D in S4 File. Complete data sets for the untreated banagrass were previously reported [34].

**Table 4:** Summary of char and ash yields from pyrolysis of pretreated banagrass.

Temp	Char_Org_	Char S.D.	Number of tests	Char_Org+Inorg_	Ash*
C	wt% daf basis		N	wt% dry basis	
400	6.4	1.3	4	9.3	2.6
450	4.8	0.9	4	7.2	2.3
500	4.4	0.5	3	7.5	2.8
600	1.5	0.5	3	4.4	2.8

Bias is estimated at ≤±2% (absolute).

Char_Org_ refers to the organic fraction of the char relative to the daf feedstock.

Char_Org+Inorg_ refers to the sum of the organic and inorganic fractions of the char relative to the dry feedstock.

* Ash refers to the ash contained within the char, given as wt% of the feedstock on a dry basis, the S.D. of the ash yield is 1.5 wt% (absolute).

The bio-oil yields follow a clear trend with the highest yields obtained at 400 to 450°C and lower yields at higher temperature, for the same vapor residence time. The permanent gas results show opposing trends to the bio-oil results with higher gas yields at higher temperatures. The amount of char_org_ (organic fraction, excluding ash) decreases with increasing temperature, from 6.4 wt% (daf) at 400°C to 1.5 wt% at 600°C.

The amount of bio-oil that is lost during rotary evaporation to remove the solvent is termed 'volatile bio-oil' in Tables A to D in S4 File. In all cases, the volatile bio-oil accounted for < 1 wt% of the products. However, the volatile bio-oil is thought to be greatly underestimated due to the extremely low concentration of bio-oil in the washing solvent (~3 mg/mL) combined with the fact that less than half the peaks that are observed are calibrated. The high variability in GCMS response factors for bio-oil compounds [42] means it is not possible to obtain a useful estimate of the concentrations of un-calibrated compounds through a comparison to the internal standard.

The results displayed in Tables A to D in S4 File also show that between 48 and 56 wt% of the starting material is not accounted for in the quantified products (labeled as 'undetected' in the tables). The undetected material is comprised of compounds that could not be quantified as they fall in a window between the species detected by the online gas analyzers (CO, CH_4_, H_2_ and CO_2_) and those quantified by the GCMS method; i.e. the undetected material includes other gases and compounds more volatile than or obscured by the GC solvent (acetone/methanol), including water and un-calibrated compounds in the GC range. This can also include compounds that are trapped in the injection port.

#### Effect of residence time

There are clear trends in the data which show higher bio-oil yields and lower gas yields are obtained as the vapor residence time decreases; which is in agreement with results reported by other researchers [38, 43]. Considering the results obtained at 400°C, the maximum bio-oil yield is ~39 wt% at the shortest residence time (RT-4, 1.5 s) with 5 wt% gas. At the second shortest (RT-3, 4.6 s) and second longest (RT-2, 8.3 s) residence times the bio-oil yields at 400°C are ~36 wt% and gas yields ~7 wt%. At the longest residence time (RT-1 12.2 s) the bio-oil yield is ~33 wt% and gas yield ~9 wt%.

#### Effect of temperature

The highest overall bio-oil yield was recorded when operating at 450°C and the shortest residence time (RT-4, 1.4 s), ~41 wt% bio-oil with ~7.5 wt% gas. The char_org_ yield at 450°C is ~4.5 wt% and at 400°C, ~6.5 wt%. The results indicate that a few extra percent of volatile material can be released from the char particles by increasing the bed temperature from 400 to 450°C with only marginally more cracking of the bio-oil vapors into permanent gases. Increasing the bed temperature to 500°C results in no further decrease in the char yield but induces more cracking of the bio-oil vapors producing more permanent gases. Increasing the bed temperature to 600°C produces even less char (~1.5 wt% char_org_), however there is much greater cracking of the bio-oil vapors which produces a significantly higher gas yield. The permanent gas data is discussed later in the manuscript.

### Results—Comparison of banagrass product yields before and after pretreatment

The product yields from the fast pyrolysis of untreated banagrass have been previously reported from experiments using the same reactor and experimental conditions as used herein [34]. Comparison of product yields before and after banagrass pretreatment shows that in all cases the bio-oil yields (daf basis, Fig 5) are higher when using the pretreated banagrass. Depending on the temperature and residence time, the bio-oil yields are between ~4 and 11 wt% (absolute) higher for the pretreated banagrass, which is considered experimentally significant.

Pretreatment of banagrass has a less significant effect on the permanent gas yields from pyrolysis (Fig 6). In almost all cases, the total amounts of permanent gases from banagrass before and after pretreatment are within experimental uncertainty of one another, for equivalent reaction conditions. For the shortest residence time experiments (BP-4), there was slightly more gas produced from the pretreated banagrass. For the char yields, any differences in the values before and after pretreatment are within experimental uncertainty and are *not* considered experimentally significant.

The values of 'undetected' material, as referred to in Tables A to D in S4 File, are always lower for the pretreated banagrass than the untreated banagrass by between ~5 to 10 wt%, which is considered to be experimentally significant and is in agreement with measured increases in the bio-oil yield.

Taken together the data strongly indicates that water pretreatment of banagrass results in higher bio-oil yields, under all the conditions examined. There was also less undetected material and approximately the same amount of char and permanent gases (or slightly more gas). This observation implies that the primary bio-oil vapors released from untreated banagrass undergo significantly more cracking as they escape the particles (intra-particle) than for the pretreated banagrass. However, this increase in bio-oil vapor cracking for untreated banagrass does not significantly increase the permanent gas yield (in some cases it slightly decreases the gas yield), instead it increases the amount of undetected material. This may well be related to changes in the amount of pyrolysis water and volatiles, although this could not be examined during the present study.

Furthermore, the data from the untreated and pretreated banagrass suggests that the bio-oil vapors that escape the particles contain different compounds. Evidence for this comes from the fact that the bio-oil from the untreated banagrass is less stable toward increased residence time or increased temperature than the equivalent bio-oils from the pretreated banagrass. For the untreated banagrass there is sharp decline in the bio-oil yield on going from the shortest residence time (RT-4) to the second shortest residence time (RT-3) at 450°C or greater; whereas the decline in bio-oil yield is less dramatic for the pre-treated banagrass. At 400°C, the bio-oil yields are between 26 and 29 wt% for the untreated banagrass over all four residence times studies; whereas for the pretreated banagrass the bio-oil yield decreases with increasing residence time (~39 wt% at BP-4, ~36 wt% at BP-3 and BP2, and ~33 wt% at BP-1).

### Results—CHN of the dried bio-oils, pretreated banagrass

**Fig 7:**
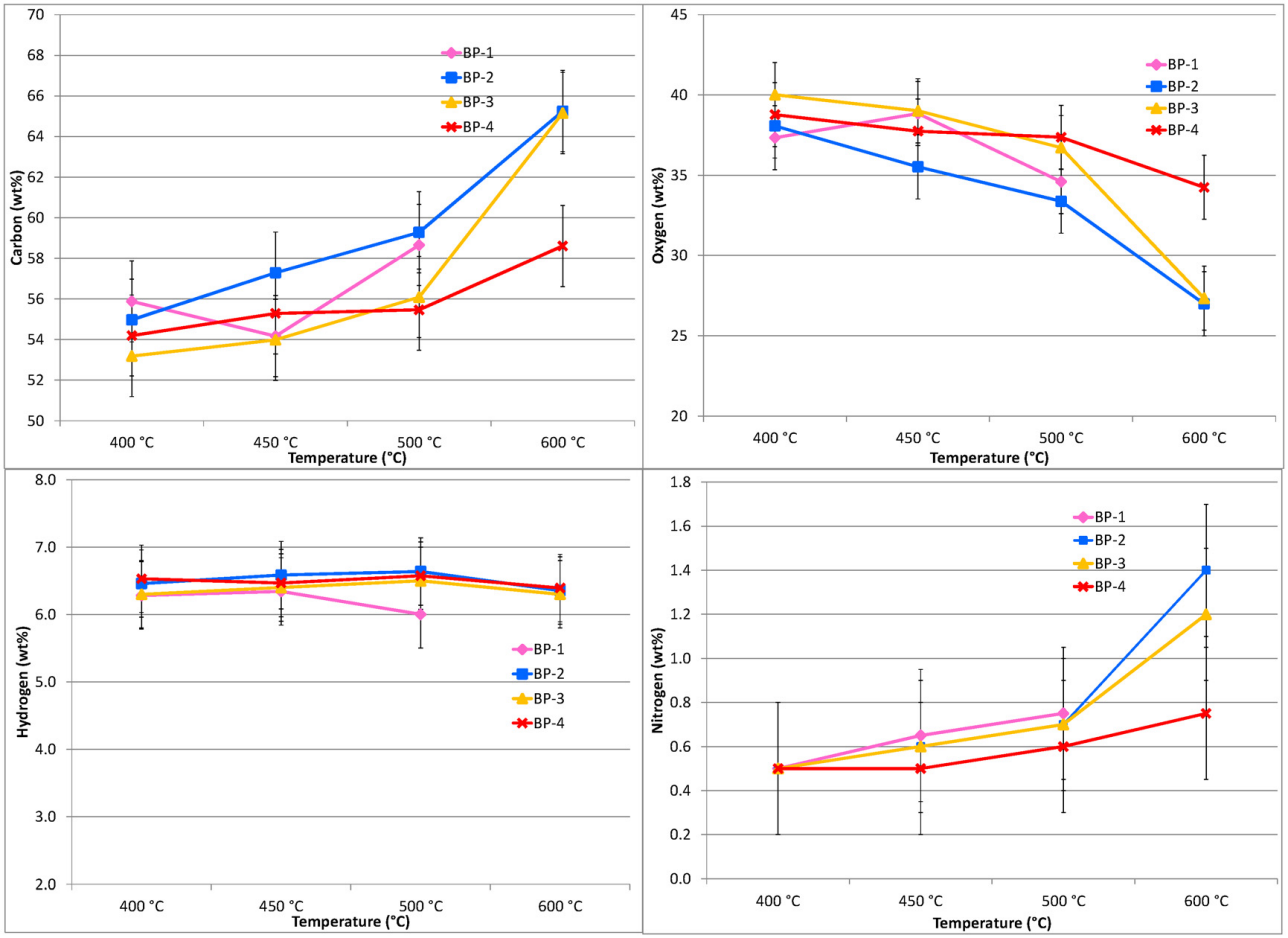
Elemental analysis (C, H, N and O by difference) results for the dried bio-oils from pyrolysis of pretreated banagrass as a function of temperature and vapor residence time. Results are presented as wt% of the **bio-oil**. The standard deviation of the C and O results is < 2.0 wt% (absolute) and for H < 0.5 wt% and N < 0.3 wt% (absolute).

**Fig 8:**
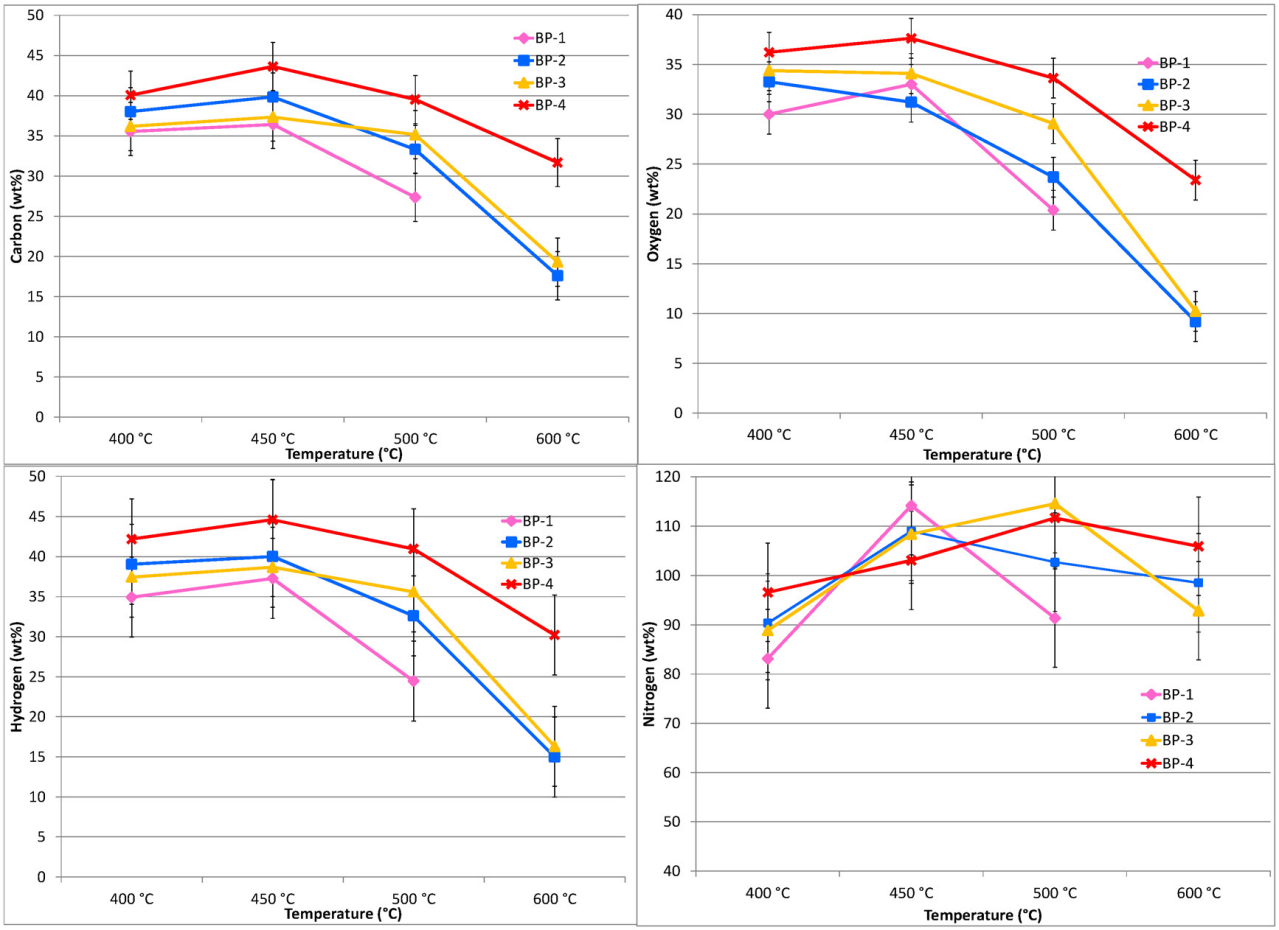
Elemental analysis (C, H, N and O by difference) results for the dried bio-oils from pyrolysis of pretreated banagrass as a function of temperature and vapor residence time. Results are presented as wt% of the **element** in the **Feedstock** (daf). The standard deviation for the C and O results is ≤ ±3.0 wt%, for H ≤ ±5.0 wt% and for N ~±10 wt% (absolute).

The quality of the bio-oils recovered at different temperatures and vapor residence times from pretreated banagrass was assessed by comparing their C, H and N contents from elemental analysis (O determined by difference). Fig 7 displays the elemental analysis results of the dry bio-oils from pretreated banagrass in terms of weight percent of C, H, N and O in the bio-oil. Fig 8 shows the same data presented relative to the element in the feedstock (daf).

The elemental analysis of the bio-oils shows the proportion of carbon increases with increasing temperature and to a lesser extent with increasing residence time (Fig 7). At 400°C there is ~53–56 wt% carbon in the bio-oil independent of residence time (i.e. differences are within experimental uncertainty). At 450°C there is 54–57 wt% carbon in the bio-oils with no clear trend due to increasing residence time. At 500°C differences start to emerge, with ~55–56 wt% carbon in the bio-oils recovered at shorter residence times (BP-3 and BP-4) and ~59 wt% at longer residence times (BP-1 and BP-2). At 600°C there is a marked increase in the amount of carbon, with ~59 wt% at the shortest residence time (BP-4), increasing to ~65 wt% at longer residence times (BP-3 and BP-2). No data is available for the longest residence time (BP-1) at 600°C as that condition was *not* examined.

The oxygen results show directly opposing trends to the carbon results which is a reflection of the values being determined by difference (Fig 7). At 400 to 450°C at the shortest residence time (BP-4) there is 36–38 wt% oxygen which decreases to ~ 30 wt% at the longest residence time (BP-1). In general, there is less oxygen in the bio-oils formed at higher temperature or longer residence time, the lowest value being ~10 wt% oxygen at 600°C. For hydrogen, there is no trend due to increasing temperature or residence time with all the values falling between 6 and 7 wt%. At 500°C there appears to be slightly less hydrogen in the bio-oil from the longest residence time (BP-1) compared to the shorter RT bio-oils. For nitrogen there is a slight trend of increasing N with increasing temperature which is more significant at longer residence times; in general there is ~0.5–0.7 wt% N across all RT's from 400 to 500°C. At 600°C, N content increases from ~0.7 wt% at the shortest residence time (BP-4) to ~1.2 wt% at second shortest RT (BP-3) and finally to ~1.4 wt% at second longest RT (BP-2).

Plotting the elemental analysis results from the bio-oil relative to the amount of the element in the feedstock (daf) shows clearer trends (Fig 8). The carbon, oxygen and hydrogen results all show the same general trends. The greatest carbon content is in the bio-oil recovered at the shortest residence time (BP-4) at 450°C, ~45 wt% C, decreasing to ~37 wt% at the longest residence time (BP-1). At 600°C, there is significantly less carbon partitioned to the bio-oils with ~32 wt% carbon at the shortest residence time (BP-4) decreasing to ~17 wt% carbon at longer residence times (BP-3 and BP-2).

The amount of oxygen in the bio-oils relative to the oxygen in the feedstock is ~34–38 wt% across the temperature range of 400 to 500°C at the shortest residence time (BP-4), with less oxygen in the bio-oils exposed to longer RT's or higher temperature (Fig 8). For hydrogen, it is ~40–45 wt% at the shortest RT at 400 to 500°C, dropping to 40–35 wt% at longer RT's. At 600°C, the H content is <30 wt% at the shortest residence time, falling to ~15 wt% at longer RT's. The nitrogen partitioned to the bio-oil is less clear due to the low absolute amount of nitrogen in the banagrass, which results in larger uncertainty. The amount of N present in the oils is typically 80–115 wt% with no clear trend due to RT, although there appears to be more N present at higher temperatures.

Comparing the bio-oil CHNO results from pretreated banagrass with equivalent data sets from the untreated banagrass [34] shows that the amounts of carbon and hydrogen in the oils are about the same, considering experimental uncertainty. The oxygen content of the bio-oils from pretreated banagrass is marginally higher or unchanged. The amount of nitrogen is slightly lower or unchanged than from untreated banagrass. Therefore, these results, when taken with yields of bio-oils relative to the feedstock, indicate that there is greater partitioning of C, H, O, and N to bio-oil after pretreatment. For example, at 450°C and the shortest residence time (BP-4) the amount of carbon in the bio-oil relative to the carbon in the pretreated banagrass is ~44 wt% and for untreated banagrass ~41 wt%. Oxygen content in the bio-oil from pretreated banagrass is ~38 wt% and from untreated banagrass ~33 wt%. This is a reflection of the greater bio-oil yields from the pretreated banagrass.

Elemental analysis was also performed on the char samples. Table 5 presents the C, H, N and O results as weight percent of the char_org_ (daf). Table 6 present the same data in terms of weight percent of the element in the feedstock (daf).

**Table 5:** Elemental analysis results for the chars (daf) from pretreated banagrass, given as wt% of the char (daf).

Temperature	C	H	N	O
C	wt%	wt%	wt%	Wt%
400	64.2	2.3	0.9	32.6
450	62.3	2.0	1.0	34.8
500	63.0	2.3	1.2	33.4
600	74.8	3.0	1.3	20.9

RSD is estimated to be <15%

**Table 6:** Elemental analysis results for the chars (daf) from pretreated banagrass, given as wt% of the element in the feedstock (daf).

Temperature	C	H	N	O
C	wt%	wt%	wt%	Wt%
400	7.8	2.4	30.3	5.0
450	5.7	1.6	23.3	4.0
500	5.3	1.7	26.0	3.6
600	2.1	0.8	10.1	0.8

RSD is estimated to be <20%

The results in Table 5 show that there is ~63–65 wt% carbon in the char_org_ (daf) at 400 to 500°C, and ~75 wt% carbon at 600°C. Hydrogen accounts for ~2 wt% at 400 to 500°C and ~3 wt% at 600°C. Nitrogen is ~1.0 wt% across all temperatures. There is ~33–35 wt% oxygen in the 400 to 500°C chars and ~20 wt% O at 600°C.

Considering the same data in terms of partitioning of elements in the char_org_ (daf) relative to the element in the feedstock (daf) shows clear decreasing trends. The amount of carbon retained by the chars decreases with increasing temperature from ~8 wt% at 400°C to ~2 wt% at 600°C. Hydrogen retention ranges from ~2.5 wt% at 400°C to ~ 1.0 wt% at 600°C. Nitrogen retention is ~30 wt% at 400°C falling to ~10 wt% at 600°C. Oxygen retention is ~5 wt% at 400°C and ~1 wt% at 600°C. For all elements, the retention is roughly the same for the 450 and 500°C chars (i.e. values are within experimental uncertainty).

Comparing the elemental analysis of the chars from the pretreated banagrass with those from the untreated banagrass [34] reveals that there is no significant differences in the weight percentage composition of carbon, hydrogen, nitrogen and oxygen. However, there are small differences when the result are considered in terms of partitioning of elements to the char. The 400°C chars from untreated banagrass retained slightly more carbon, hydrogen and oxygen than the chars from pretreated banagrass, whereas nitrogen retention was slightly less for the untreated chars. At higher temperatures the retention of carbon, hydrogen and oxygen is roughly the same for the chars from untreated and pretreated banagrass; whereas, there appears to be more retention for nitrogen in the char from the pretreated samples.

### Results—Permanent gas data, pretreated banagrass

**Fig 9:**
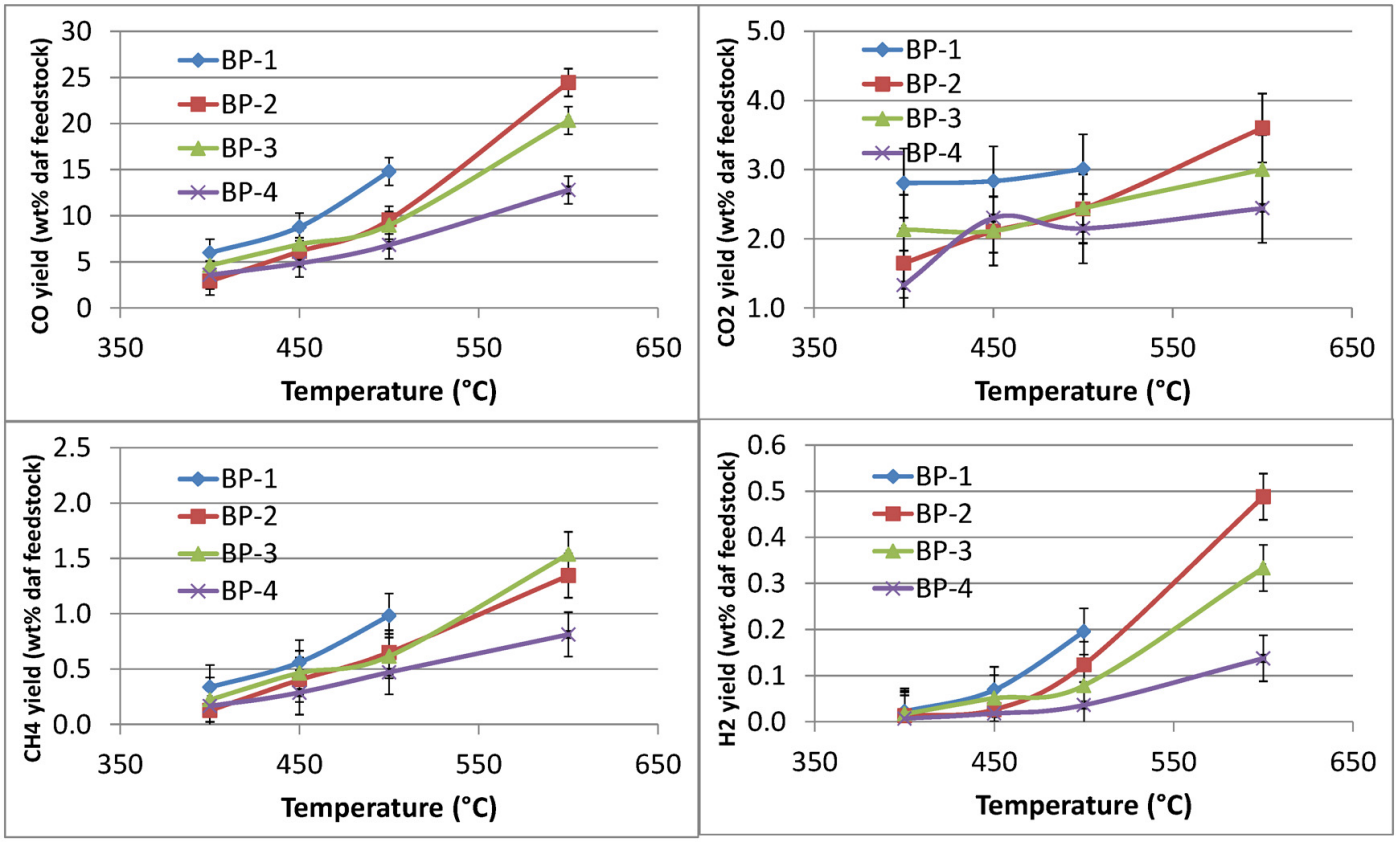
Permanent gas data (CO, CO_2_, CH_4_ and H_2_) from the pyrolysis of banagrass S3 as a function of temperature and vapor residence time, presented as wt% of the daf feedstock (BP, bed position). The standard deviation for the CO values is ≤ ±1.5 wt% (absolute), for CO_2_ ≤ ±0.5 wt%, for CH_4_ ≤ ±0.2 wt% and for H_2_ ≤ ±0.05 wt%.

The permanent gas data from the pyrolysis tests are reported in terms of wt% of CO, CO_2_, CH_4_ and H_2_ relative to the dry ash free feedstock in Fig 9; the same data are tabulated in Tables A to D in S5 File. The permanent gas data were obtained from online gas analyzers with associated measures of uncertainty as explained in the experimental section. The results should be considered as indicative. Nonetheless, the repeatability of the results was good, see Fig 9.

Fig 9 shows that the amounts of CO, CO_2_, CH_4_ and H_2_ generated during pyrolysis gradually increases with increasing temperature from 400 to 500°C with more significant increases across the 500 to 600°C range. There is also a trend of increasing amounts of gases as volatiles residence time increases, with small increases associated with increasing the residence time from BP-4 to BP-3 to BP-2 and then a more significant increasing residence time from BP-2 to the longest BP-1 tests.

CO increased from ~4 wt% at 400°C at the shortest residence time (BP-4) to ~6 wt% at the longest RT (BP-1). At 450°C the amount of CO increased from ~5 wt% at shortest RT (BP-4) to ~9 wt% at the longest RT. At 500°C the amount of CO increases from ~7 wt% at the shortest RT to ~15 wt% at the longest RT. At 600°C the amount of CO increases from ~13 wt% at the shortest RT to ~25 wt% at the second longest RT (note: no data are available for the longest RT at 600°C).

With the exception of the lowest pyrolysis temperature (400°C), methane produced in the pyrolysis process generally increases as a function of temperature and residence time. Less than 0.5 wt% methane was produced at 400°C across all residence times. At 450°C the amount of CH_4_ increases from ~0.3 wt% at the shortest RT to ~0.6 wt% at the longest RT. At 500°C there is ~0.5 wt% CH_4_ at the shortest RT, increasing to ~1.0 wt% at the longest RT. At 600°C the amount of CH_4_ increase from ~0.8 wt% at the shortest RT to ~1.5 wt% at the second longest RT.

Very little H_2_ (< 0.1 wt%) is generated at temperatures below 500°C across all residence times. At 500°C, H_2_ increases from ~0.05 wt% at the shortest RT to ~0.20 wt% at the longest RT. More H_2_ is formed at 600°C, with ~0.15 wt% at the shortest RT and ~0.50 wt% at the second longest RT.

The production of CO_2_ in the pyrolysis process was found to fall within a range of 1.5 to 3.5 wt% of fuel mass over the range of temperatures and residence times, with a general increasing trend with increasing reaction temperature and residence time.

Comparing the permanent gas data from the untreated [34] and pretreated banagrass shows that for CO there is no significant difference in the results over the temperature range 400 to 500°C and across all residence times. Differences start to emerge for the longest RT data sets at 500°C with ~12 wt% CO from the untreated banagrass and ~15 wt% for the pretreated banagrass which is considered to be experimentally significant. At 600°C the amount of CO is slightly greater from the pretreated banagrass than the untreated banagrass, by ~2–4 wt% at each residence time. For CH_4_, the results are roughly the same from the untreated and pretreated banagrass, i.e. within experimental uncertainty; likewise for the H_2_ results. For CO_2_, in general, all the values are within experimental uncertainty with the exception of the 600°C results, where there is slightly more CO_2_ generated from the untreated banagrass than the pretreated banagrass (by ~1–2 wt%) at all residence times.

In summary, there is slightly more CO and less CO_2_ generated during the pyrolysis of pretreated banagrass than the untreated banagrass at temperatures ≥ 500°C, and no significant differences at temperatures < 500°C.

### Results—Pyrolysis products from the other tropical biomasses

Results are presented below from the fast pyrolysis of eucalyptus, leucaena, sugarcane bagasse, energy cane and pretreated energy cane (S3) at a single reaction condition (450°C and 1.4 s residence time) for comparison to the banagrass results. Table 7 presents pyrolysis product yields in terms of bio-oil, char and permanent gases. Table 8 gives a summary of the char and ash yields. Table 9 provides a breakdown on the permanent gases in terms of wt% of CO, CO_2_, CH_4_ and H_2_ relative to the amount of feedstock (daf).

**Table 7:** Bio-oil, char and gas yields (wt% feedstock daf) from pyrolysis of eucalyptus, leucaena, sugarcane bagasse, energy cane, pretreated energy cane (S3), banagrass and pretreated banagrass (S3) at the shortest residence time (BP-4).

Sample	Temperature	Dry bio-oil^¥^	Volatile bio-oil^#^	Char_org_*	^CO CO_2_ CH_4_ H_2_	Undetected**
	°C	wt%	wt%	wt%	wt%	wt%
Eucalyptus	450	48.1	0.1	4.2	6.8	40.8
Leucaena	450	40.8	0.3	2.6	6.5	49.9
S-Bagasse	450	55.1	<LLQ	2.2	5.6	37.1
E-Cane	450	46.8	0.2	3.7	6.3	42.9
E-Cane S3	450	55.3	<LLQ	3.5	6.1	35.1
Banagrass	450	36.7	0.2	3.0	7.8	52.5
Banagrass S3	450	41.3	<LLQ	3.5	7.4	47.8

^¥^ S.D. of the 'dry bio-oil' yield is < ±2.0 wt% (absolute).

^#^ Volatile bio-oil refers to the amount of bio-oil removed from the sample during drying and is determined by analyzing the bio-oil solution by GCMS before drying and again after it is dried.

<LLQ, less than the lower limit of quantification, which equates to a yield of less than 2.0 wt% of the 'daf' feedstock.

^ Indicative values derived from on-line gas analysis.

* The bias in the char yield is estimated to be < ±2% (absolute) and S.D. < ±1.5 wt%, see Table 8 for the amount of ash contained within the char.

** 'Undetected' is derived as: 100%—(Dry bio-oil + Volatile bio-oil + Char + CO, CO_2_, CH_4_ and H_2_ yields).

**Table 8:** Summary of char and ash yields (wt% feedstock) from fast pyrolysis of eucalyptus, leucaena, sugarcane bagasse, energy cane, pretreated energy cane (S3), banagrass and pretreated banagrass (S3).

Sample	Temperature	Char_Org_	Char_Org+Inorg_	Ash*	Feedstock Ash
	°C	wt% daf	wt% db		wt% db
Eucalyptus	450	4.2	5.1	0.9	0.7
Leucaena	450	2.6	3.1	0.5	1.5
S-Bagasse	450	2.2	6.3	3.9	7.6
E-Cane	450	3.7	7.1	3.2	6.6
E-Cane S3	450	3.5	4.8	1.2	3.2
Banagrass^#^	450	3.9	8.1	3.9	8.5
Banagrass S3^#^	450	4.6	7.1	2.3	5.1

Bias in the char yield is estimated at ≤ ±2% (absolute) and S.D. < ±1.5 wt%.

* Ash refers to the ash contained within the char, given as wt% of the feedstock on a dry basis, the S.D. of the ash yield is < ±1.5 wt% (absolute).

Char_Org_ refers to the organic fraction of the char relative to the daf feedstock.

Char_Org+Inorg_ refers to the sum of the organic and inorganic fractions of the char relative to the dry feedstock.

^#^ The results for banagrass are the average of 4 tests, whereas the other samples are based on a single result.

**Table 9:** Permanent gas data (wt% feedstock daf) from the pyrolysis of eucalyptus, leucaena, sugarcane bagasse, energy cane, pretreated energy cane (S3), banagrass and pretreated banagrass (S3) at the shortest residence time (BP-4) and 450°C.

Sample	Temperature	CO	CO_2_	CH_4_	H_2_	Total Producer Gas
	C	wt%	wt%	Wt%	wt%	L/g daf
Eucalyptus	450	5.0	1.3	0.4	0.02	0.06
Leucaena	450	4.1	2.1	0.3	0.01	0.05
S-Bagasse	450	3.8	1.5	0.3	0.01	0.05
E-Cane	450	4.0	2.0	0.3	0.01	0.05
E-Cane S3	450	4.1	1.7	0.3	0.01	0.05
Banagrass	450	5.2	2.3	0.3	0.01	0.06
Banagrass S3	450	4.8	2.3	0.3	0.02	0.06

S.D. for the CO values is ≤ ±1.5 wt% (absolute), for CO_2_ ≤ ±0.5 wt%, for CH_4_ ≤ ±0.2 wt% and for H_2_ ≤ ±0.05 wt%.

The results in Table 7 are presented in order of decreasing yield in Table 10. The greatest dry bio-oil yields are from pretreated energy cane (S3) and sugarcane bagasse (~55 wt%) followed by eucalyptus and energy cane (~48 wt%). The greatest char yields are from eucalyptus, energy cane and pretreated energy cane. The results for eucalyptus in Tables 7 and 10 do not match literature, i.e. eucalyptus typically produces a high bio-oil yield (organic fraction ~50–60 wt% daf) and relatively low amounts of char [2–5]. The slightly low bio-oil and high char yields for eucalyptus may be due to the reaction temperature used herein (450°C) compared to literature sources (~500°C). A detailed account of the char yields is given in Table 8.

**Table 10:** Products from fast pyrolysis of the various feedstock's, shown in order of decreasing yield, based on the data in Tables 7 and 9.

Product	Order of decreasing yield by feedstock
Bio-oil yield	E-cane S3 = S-bag > Eucalyptus ≥ E-cane >> Leucaena = Bana S3 >> Bana
Char yield*	Eucalyptus ≥ E-Cane = E-Cane S3 = BanaS3 > Bana > Leucaena > S-bag
Gas yield	Bana ≥ Bana S3 > Eucalyptus ≥ Leucaena ≥ E-Cane ≥ E-Cane S3 ≥ S-bag
CO yield	Bana ≥ Eucalyptus ≥ Bana S3 > Leucaena = E-Cane S3 ≥ E-Cane ≥ S-bag
CO2 yield	Bana = Bana S3 ≥ Leucaena ≥ E-Cane > E-Cane S3 > S-bag > Eucalyptus
Undetected	Bana > Leucaena > Bana S3 > E-Cane > Eucalyptus > S-Bag > E-Cane S3

* The trend for char_org_ yield is based on the results in Table 7 which are from a single experiment with the bed in its highest position. Char recovery is more difficult when working with the bed in its highest position and always results in an underestimation when compared to char yields from lower bed positions. The banagrass char yields shown in Table 8 are the average of tests at each bed position (4 tests) which results in higher values than in Table 7. Considering the S.D. all the samples generate the same amount of char expect for sugarcane bagasse and leucaena which produce less.

Table 8 shows that all the samples generate ~4 wt% char_org_ except for leucaena and sugarcane bagasse which produced ~2.0 to 2.5 wt% char_org_. The standard deviation of the char values is < ±1.5 wt% (absolute). The ash results show that most, if not all, the ash from eucalyptus and leucaena is retained in the char (within experimental uncertainty), while for the other fuels about half the ash is retained within the char.

The greatest permanent gas yield is from untreated banagrass followed by pretreated banagrass and the hardwoods. Pretreated energy cane and sugarcane bagasse produced the least gas (Tables 7 and 10). Note however, that almost all differences between the gas yield values are within experimental uncertainty. The amounts of CO and CO_2_ produced rather than the total amount of product gas (9 and 10) are better indicators of performance. Banagrass produces the most CO followed by eucalyptus and pretreated banagrass, with energy cane and sugarcane bagasse yielding the least. CO_2_ production is the highest for pretreated and untreated banagrass followed by leucaena. Sugarcane bagasse and eucalyptus were observed to generate the least CO_2_.

The amounts of 'undetected' material (Tables 7 and 10) in the pyrolysis products of the seven biomass materials were greatest for untreated banagrass and leucaena, and lowest for eucalyptus, sugarcane bagasse and pretreated energy cane. The undetected material provides an indicator of the combined amount of pyrolysis water and volatiles in the bio-oil, products that were not quantified in the analysis.

In summary, the data in Tables 7 to 10 shows that based on the yields of 'dry bio-oil', CO and CO_2_, sugarcane bagasse, pretreated energy cane and eucalyptus were the best feedstocks for fast pyrolysis. On the same basis, the worst feedstocks were untreated banagrass followed by pretreated banagrass and leucaena. This ranking system placed untreated energy cane in the middle of the grouping.

Tables 11 and 12 display the elemental analysis results for the dry bio-oils and chars, respectively, from fast pyrolysis of eucalyptus, leucaena, sugarcane bagasse, energy cane, pretreated energy cane (S3), banagrass and pretreated banagrass (S3) at the shortest residence time (BP-4, 1.4 s) and 450°C.

**Table 11:** Elemental analysis results for the dry bio-oil from eucalyptus, leucaena, sugarcane bagasse, energy cane, energy cane S3, banagrass and banagrass S3 at the shortest residence time (BP-4) and 450°C. Presented as wt% of the element in the feedstock (daf).

Sample	C	H	N	O
	wt%	wt%	wt%	wt%
Eucalyptus	51.0	52.1	117.0	44.1
Leucaena	46.3	45.1	109.1	33.6
S-Bagasse	56.4	61.3	52.6	52.6
E-Cane	50.1	52.3	50.5	42.5
E-Cane S3	55.4	59.7	86.8	54.3
Banagrass	40.8	43.6	58.3	32.8
Banagrass S3	43.6	44.6	103.1	37.6

The standard deviation for the C and O results is ≤ ±3.0 wt%, for H ≤ ±5.0 wt% and for N ~±20 wt% (absolute).

**Table 12:** Elemental analysis results for the char_org_ (daf) from eucalyptus, leucaena, sugarcane bagasse, energy cane, energy cane S3, banagrass and banagrass S3 at the shortest residence time (BP-4) and 450°C. Presented as wt% of the element in the feedstock (daf).

Sample	C	H	N	O
	wt%	wt%	wt%	wt%
Eucalyptus	6.2	1.8	16.6	2.1
Leucaena	3.5	1.1	10.2	1.7
S-Bagasse	3.1	1.4	6.1	1.2
E-Cane	5.5	1.7	15.8	1.8
E-Cane S3	4.2	1.2	11.0	2.9
Banagrass	5.0	1.8	7.9	2.9
Banagrass S3	5.7	1.6	23.3	4.0

RSD is estimated to be <20%

The results in Table 11 show the partitioning of carbon, hydrogen, nitrogen and oxygen in the bio-oils relative to the element in the feedstock (daf) and overall trends are similar to the trend observed in dry bio-oil yields. Sugarcane bagasse, pretreated energy cane and eucalyptus have the highest C partitioning (~51–56 wt%), whereas banagrass and pretreated banagrass (~40–44 wt%) defined the lower end of the range. Oxygen partitioning followed a similar trend to that of carbon, with bio-oil from sugarcane bagasse and pretreated energy cane containing ~50–55 wt% of the feedstock oxygen, with the lowest amounts, 33 wt%, determined for banagrass and leucaena bio-oils. Hydrogen partitioning to bio-oil was greatest for sugarcane bagasse and pretreated energy cane (~60 wt%) and lowest for banagrass and pretreated banagrass (~44 wt%). Data for nitrogen are less conclusive due to the small amounts of nitrogen present in the feedstocks which resulted in larger measurement uncertainties. Despite this limitation, there appears to be a greater partitioning of nitrogen to the bio-oils from eucalyptus, leucaena, pretreated banagrass and pretreated energy cane than for the other feedstocks.

Comparison of the feedstocks that had untreated and pretreated samples shows that in all cases there is greater partitioning of all elements to the bio-oil from the pretreated samples. The effect appears to be more significant for nitrogen than other elements but this may be a reflection of the greater uncertainty in the nitrogen results.

The partitioning of elements to the char product is fairly low in all cases, with values for C < 7 wt%, H < 2 wt%, N < 20 wt% and O < 4 wt% (Table 12). The chars from eucalyptus, pretreated banagrass and untreated energy cane appear to retain slightly more carbon (~6 wt%) than the other feedstocks, with the lowest retention of carbon (~3.0–3.5 wt%) in the chars from sugarcane bagasse and leucaena. The char from pretreated banagrass appears to retain the most oxygen (~4 wt%) followed by banagrass and pretreated energy cane (~3 wt%).

### Results—Comparison to literature

As noted in the introduction, various correlations between feedstock properties and fast pyrolysis product yields have been reported [2, 4, 5, 9, 12]. Correlations between bio-oil yields and feedstock properties include: the amounts of ash, Na + K, AAEM, volatile matter, and hemi-cellulose, and the O/C wt./wt. ratio of the feedstock. Based on the data in the previous sections, Table 13 summarizes the predicted trends for bio-oil yields based on the literature correlations, along with the trend determined from measured bio-oil yields.

**Table 13:** Predicted bio-oil yields from fast pyrolysis based on correlations to feedstock properties as reported in literature [2, 4, 5, 9, 12].

Basis	Predicted trend
Ash	Eucalyptus > Leucaena > E-Cane S3 > Bana S3 > E-Cane > S-bag >> Bana
Na + K	Eucalyptus > S-bag = E-Cane S3 > Leucaena > Bana S3 >> E-Cane >> Bana
AAEM	E-Cane S3 > Eucalyptus > S-bag > Bana S3 > Leucaena >> E-Cane >> Bana
Volatiles	E-Cane S3 = Eucalyptus > Bana S3 > Bana = Leucaena > S-Bag > E-Cane
O/C (wt/wt)	Woods: Leucaena = Eucalyptus
O/C (wt/wt)	Grasses: E-Cane > Bana > S-Bag > Bana S3 > E-cane S3
Cellulose	Eucalyptus ≥ Leucaena ≥ S-Bagasse > E-cane = Bana S3 ≥ E-Cane S3 ≥ Bana
Lignin	Leucaena ≥ E-Cane S3 ≥ Eucalyptus > S-Bag > Bana ≥ E-cane ≥ Bana S3
Hemi-cellulose	S-Bag > Bana S3 ≥ Bana ≥ E-Cane S3 > E-cane > Leucaena > Eucalyptus
*Actual Yields*	*E-cane S3 = S-bag > Eucalyptus* ≥ *E-cane >> Leucaena = Bana S3 >> Bana*

The actual trend in bio-oil yields is in closest agreement with the yields predicted by volatile matter content of the feedstock with the exception of sugarcane bagasse which is predicted to produce less bio-oil than was observed experimentally. The trend based on amounts of Na + K was also similar to the measured trend, although the energy cane experimental yield of bio-oil was greater than predicted. Using AAEM as a predictor of bio-oil yield did not completely agree with measured values; energy cane was predicted to produce less bio-oil than leucaena. The total amount of ash and O/C ratio in the feedstock both proved to be poor indicators of bio-oil yield. Differences in the O/C ratios between feedstock's was relatively small even when both woods and grasses were included (woods ~0.85–0.90 and grasses ~0.80–0.85, Table 2). This finding differs from the results reported by Oasmaa et al. [2] where straws displayed O/C ratios of ~0.95–1.05 and woods ~0.80–0.85. Finally, the percentages of cellulose, lignin and hemi-cellulose do not appear to be useful predictors of bio-oil yields from the feedstocks.

The above predictions show that trying to draw simple correlations between the feedstock properties and pyrolysis bio-oil yields can be confusing or contradictory. It appears from the results and trends shown in Tables 7, 10 and 13, and the literature, that the weight percentages of volatile matter and AAEM are currently the most useful indicators of bio-oil yield [2, 5, 9, 12]. The actual bio-oil yield is however dependent on the combined effects of several of the above mentioned variables and it is not entirely clear which, if any, of these properties are dominate.

Based on the data, the effects of pretreatment (i.e. reducing the amount of ash and AAEM) of banagrass and energy cane on pyrolysis products include: 1) an increase in the amount of dry bio-oil, 2) a decrease in the amount of undetected material, 3) little or no change in char yield, 4) no effect on the total gas yield, 5) a slight increase, if any, in CO yield, and 6) a slight decrease or no change in CO_2_ yield. These findings are in general agreement with those reported in literature [2, 5, 9, 11, 12]. There are, however, some discrepancies. Oasmaa [2] found that decreasing amounts of AAEM resulted in less permanent gases being formed, whereas Fahmi [12] reported the opposite, and Mourant [5] saw no effect which matches the findings reported here. Note: Oasmaa compared different types of biomass, whereas Fahmi and Mourant compared samples before and after pretreatment. Fahmi [11] also found that the char yields increased with increasing AAEM content which differs from results reported here.

There is indirect evidence from the current study that bio-oils from pretreated banagrass are more stable (less aging reactions) than the bio-oils from untreated banagrass produced at identical reaction conditions. The evidence comes from GCMS analysis of the bio-oils (see S2 File). For the untreated banagrass, very high concentrations of 2, 2-dimethoxypropane were present in the bio-oils (5 to 80 wt% relative to the feedstock daf, at 400 to 600°C respectively) and the amount increased significantly with increasing storage time [34]. For the pretreated banagrass, the bio-oils contain less 2, 2-dimethoxypropane (~2 to 15 wt% at 400 to 600°C respectively) and the amount increased less rapidly during storage. Our data suggests that 2, 2-dimethoxypropane is a product of reactions between the bio-oil and the solvent (mixture of acetone and methanol) used to recover the bio-oil from the traps, which matches the findings of other researchers [44]. This evidence for a more stable bio-oil from pretreated banagrass matches literature findings where biomass species with higher ash contents produce a less stable bio-oil [2, 12].

## Conclusions

The fast pyrolysis behaviour of pretreated banagrass was examined across a range of temperatures (400 to 600°C) and vapor residence times (~1.2 to 12 s). The greatest yield of bio-oil was obtained when working with the shortest residence time (1.4 s) at 450°C, ~41 wt% dry bio-oil with ~7.5 wt% gas and ~4.5 wt% char_org_ relative to the feedstock (daf). The dry bio-oil produced under these conditions contains ~55 wt% C relative to the bio-oil, ~6.5 wt% H, ~0.5 wt% N and ~38 wt% O. Element partitioning to the bio-oil relative to their occurrence in the feedstock (daf) was determined to be ~45 wt% for C, ~45 wt% for H, ~100 wt% for N and ~16 wt% for O. The permanent gases were dominated by CO (~5 wt% relative to feedstock daf) and CO_2_ (~2.3 wt%).

Previously, untreated banagrass was examined using the same reactor system and reaction conditions as used herein [34]. Comparing the dry bio-oil yields from the untreated and pretreated banagrass showed that the yields were greater from the pretreated sample by 4 to 11 wt% (absolute) across all test conditions. The same was found for pretreated energy cane. There appears to be no significant difference in the char yields due to pretreatment, or the total gas yields. There is, however, possibly slightly more CO and less CO_2_ generated during the pyrolysis of pretreated banagrass than the untreated banagrass at pyrolysis temperatures ≥ 500°C, or no significant difference at temperatures < 500°C. For energy cane the same trend was observed, although the differences in CO and CO_2_ yields before and after pretreatment were smaller than the experimental uncertainty.

Test results from eucalyptus, leucaena, sugarcane bagasse, energy cane and pretreated energy cane, indicated that the greatest dry bio-oil yields were from sugarcane bagasse and pretreated energy cane (~55 wt% relative to feedstock daf) followed by eucalyptus and untreated energy cane (~47–48 wt%). The total amounts of permanent gases were similar from all the feedstocks; banagrass and pretreated banagrass produce the most (~7.5–8.0 wt% relative to feedstock daf) followed by eucalyptus and leucaena (~6.5–7.0 wt%). Yields of CO are also similar from all feedstocks, with the highest yields observed from banagrass, pretreated banagrass and eucalyptus (~5 wt% relative to feedstock daf) while all the other feedstock's produce ~4 wt%. Banagrass and pretreated banagrass yielded ~2.3 wt% CO_2_ with ~1.5 wt% produced from the other feedstock's. Char_org_ yields (daf) were the lowest for sugarcane bagasse and leucaena (~2.5 wt% relative to feedstock daf) whereas the remaining feedstocks produced ~4 wt% char_org_.

Of the materials tested, the best feedstocks for fast pyrolysis were sugarcane bagasse, pretreated energy cane and eucalyptus based on the yields of 'dry bio-oil', CO and CO_2_. On the same basis, the least productive feedstock's are untreated banagrass followed by pretreated banagrass and leucaena.

Based on these results, the effect of pretreating banagrass and energy cane (i.e. reducing the amount of ash and AAEM) on pyrolysis products is: 1) to increase the dry bio-oil yield, 2) to decrease the amount of undetected material, 3) to produce a slight increase in CO yield or no change, 4) to slightly decrease CO_2_ yield or no change, and 5) to produce a more stable bio-oil (less aging). Char yield and total gas yield were unaffected by feedstock pretreatment. These findings are in general agreement with those reported in the literature [2, 5, 9, 11, 12].

## Supporting Information

S1 File Temperature distributions across the bed and freeboard. (DOC)

S2 File GCMS results. (DOC)

S3 File Elemental analysis of the biomass ashes—Tabulated. (DOC)

S4 File Fast pyrolysis product yields (bio-oil, char and gas)—Tabulated. (DOC)

S5 File Permanent gas results—Tabulated. (DOC)

## References

[1] Hawaii.gov. Available: http://www.hawaiicleanenergyinitiative.org. Accessed 31 March 2015.

[2] Oasmaa A, Solantausta Y, Arpiainen V, Kuoppala E, Sipilä K. Fast Pyrolysis Bio-Oils from Wood and Agricultural Residues. Energy & Fuels. 2010;24(2):1380–8. 10.1021/ef901107f

[3] Joubert J-E, Carrier M, Dahmen N, Stahl R, Knoetze JH. Inherent process variations between fast pyrolysis technologies: A case study on Eucalyptus grandis. Fuel Processing Technology. 2015;131(0):389–95. 10.1016/j.fuproc.2014.12.012.

[4] Kim KH, Kim T-S, Lee S-M, Choi D, Yeo H, Choi I-G, et al Comparison of physicochemical features of biooils and biochars produced from various woody biomasses by fast pyrolysis. Renewable Energy. 2013;50(0):188–95. 10.1016/j.renene.2012.06.030.

[5] Mourant D, Wang Z, He M, Wang XS, Garcia-Perez M, Ling K, et al Mallee wood fast pyrolysis: Effects of alkali and alkaline earth metallic species on the yield and composition of bio-oil. Fuel. 2011;90(9):2915–22. 10.1016/j.fuel.2011.04.033.

[6] Morgan TJ, Kandiyoti R. Pyrolysis of Coals and Biomass: Analysis of Thermal Breakdown and Its Products. Chemical Reviews. 2014;114(3):1547–607. 10.1021/cr400194p 24160484

[7] Heidari A, Stahl R, Younesi H, Rashidi A, Troeger N, Ghoreyshi AA. Effect of process conditions on product yield and composition of fast pyrolysis of Eucalyptus grandis in fluidized bed reactor. Journal of Industrial and Engineering Chemistry. 2014;20(4):2594–602. 10.1016/j.jiec.2013.10.046.

[8] Keffer VI, Turn SQ, Kinoshita CM, Evans DE. Ethanol technical potential in Hawaii based on sugarcane, banagrass, Eucalyptus, and Leucaena. Biomass and Bioenergy. 2009;33(2):247–54. 10.1016/j.biombioe.2008.05.018.

[9] Greenhalf CE, Nowakowski DJ, Harms AB, Titiloye JO, Bridgwater AV. A comparative study of straw, perennial grasses and hardwoods in terms of fast pyrolysis products. Fuel. 2013;108(0):216–30. 10.1016/j.fuel.2013.01.075.

[10] Boateng AA, Daugaard DE, Goldberg NM, Hicks KB. Bench-Scale Fluidized-Bed Pyrolysis of Switchgrass for Bio-Oil Production†. Industrial & Engineering Chemistry Research. 2007;46(7):1891–7. 10.1021/ie0614529

[11] Fahmi R, Bridgwater AV, Thain SC, Donnison IS, Morris PM, Yates N. Prediction of Klason lignin and lignin thermal degradation products by Py—GC/MS in a collection of Lolium and Festuca grasses. Journal of Analytical and Applied Pyrolysis. 2007;80(1):16–23. 10.1016/j.jaap.2006.12.018.

[12] Fahmi R, Bridgwater AV, Donnison I, Yates N, Jones JM. The effect of lignin and inorganic species in biomass on pyrolysis oil yields, quality and stability. Fuel. 2008;87(7):1230–40. 10.1016/j.fuel.2007.07.026.

[13] Montoya JI, Valdés C, Chejne F, Gómez CA, Blanco A, Marrugo G, et al Bio-oil production from Colombian bagasse by fast pyrolysis in a fluidized bed: An experimental study. Journal of Analytical and Applied Pyrolysis. 2015;112(0):379–87. 10.1016/j.jaap.2014.11.007.

[14] Islam MR, Haniu H, Islam MN, Uddin MS. Thermochemical Conversion of Sugarcane Bagasse into Bio-Crude Oils by Fluidized-Bed Pyrolysis Technology. Journal of Thermal Science and Technology. 2010;5(1):11–23. 10.1299/jtst.5.11

[15] Fraga A-R, Gaines AF, Kandiyoti R. Characterization of biomass pyrolysis tars produced in the relative absence of extraparticle secondary reactions. Fuel. 1991;70(7):803–9. 10.1016/0016-2361(91)90186-E.

[16] Xu R, Ferrante L, Briens C, Berruti F. Bio-oil production by flash pyrolysis of sugarcane residues and post treatments of the aqueous phase. Journal of Analytical and Applied Pyrolysis. 2011;91(1):263–72. 10.1016/j.jaap.2011.03.001.

[17] Phan BMQ, Duong LT, Nguyen VD, Tran TB, Nguyen MHH, Nguyen LH, et al Evaluation of the production potential of bio-oil from Vietnamese biomass resources by fast pyrolysis. Biomass and Bioenergy. 2014;62(0):74–81. 10.1016/j.biombioe.2014.01.012.

[18] Ogoshi R. Crop assessment Task B1 Report submitted to Black and Veatech. HNEI, 2008.

[19] Tran N, Illukpitiya P, Yanagida JF, Ogoshi R. Optimizing biofuel production: An economic analysis for selected biofuel feedstock production in Hawaii. Biomass and Bioenergy. 2011;35(5):1756–64. 10.1016/j.biombioe.2011.01.012.

[20] Yoshida T, Turn SQ, Yost RS, Antal MJ. Banagrass vs Eucalyptus Wood as Feedstocks for Metallurgical Biocarbon Production†. Industrial & Engineering Chemistry Research. 2008;47(24):9882–8. 10.1021/ie801123a

[21] http://www.bladeenergy.com/Bladepdf/Blade-Switchgrass-Mgmt_2ed.pdf. Accessed 21 April 2015.

[22] Turn SQ, Kinoshita CM, Ishimura DM, Zhou J. The fate of inorganic constituents of biomass in fluidized bed gasification. Fuel. 1998;77(3):135–46. 10.1016/S0016-2361(97)00190-7.

[23] Vassilev SV, Baxter D, Andersen LK, Vassileva CG, Morgan TJ. An overview of the organic and inorganic phase composition of biomass. Fuel. 2012;94(Copyright (C) 2012 American Chemical Society (ACS). All Rights Reserved.):1–33. 10.1016/j.fuel.2011.09.030

[24] Mesa-Pérez JM, Cortez LAB, Marín-Mesa HR, Rocha JD, Peláez-Samaniego MR, Cascarosa E. A statistical analysis of the auto thermal fast pyrolysis of elephant grass in fluidized bed reactor based on produced charcoal. Applied Thermal Engineering. 2014;65(1–2):322–9. 10.1016/j.applthermaleng.2013.12.072.

[25] Braga R, Costa T, Freitas JO, Barros JF, Melo DA, Melo MF. Pyrolysis kinetics of elephant grass pretreated biomasses. J Therm Anal Calorim. 2014;117(3):1341–8. 10.1007/s10973-014-3884-2

[26] Mythili R, Venkatachalam P, Subramanian P, Uma D. Characterization of bioresidues for biooil production through pyrolysis. Bioresource Technology. 2013;138(0):71–8. 10.1016/j.biortech.2013.03.161. 23612164

[27] Lee M-K, Tsai W-T, Tsai Y-L, Lin S-H. Pyrolysis of napier grass in an induction-heating reactor. Journal of Analytical and Applied Pyrolysis. 2010;88(2):110–6. 10.1016/j.jaap.2010.03.003.

[28] Andersen LK, Morgan TJ, Boulamanti AK, Álvarez P, Vassilev SV, Baxter D. Quantitative X-ray Fluorescence Analysis of Biomass: Objective Evaluation of a Typical Commercial Multi-Element Method on a WD-XRF Spectrometer. Energy & Fuels. 2013;27(12):7439–54. 10.1021/ef4015394

[29] Morgan TJ, George A, Boulamanti AK, Álvarez P, Adanouj I, Dean C, et al Quantitative X-ray Fluorescence Analysis of Biomass (Switchgrass, Corn Stover, Eucalyptus, Beech, and Pine Wood) with a Typical Commercial Multi-Element Method on a WD-XRF Spectrometer. Energy & Fuels. 2015;29(3):1669–85. 10.1021/ef502380x

[30] George A, Morgan TJ, Kandiyoti R. Pyrolytic Reactions of Lignin within Naturally Occurring Plant Matrices: Challenges in Biomass Pyrolysis Modeling Due to Synergistic Effects. Energy & Fuels. 2014;28(11):6918–27. 10.1021/ef501459c

[31] Shafizadeh F. Pyrolytic Reactions and Products of Biomass. In: Overend RP, Milne TA, Mudge LK, editors. Fundamentals of Thermochemical Biomass Conversion: Springer Netherlands; 1985 p. 183–217.

[32] Liden AG, Berruti F, Scott DS. A Kinetic Model For The Production Of Liquids From The Flash Pyrolysis Of Biomass. Chemical Engineering Communications. 1988;65(1):207–21. 10.1080/00986448808940254

[33] Strezov V, Evans TJ, Hayman C. Thermal conversion of elephant grass (Pennisetum Purpureum Schum) to bio-gas, bio-oil and charcoal. Bioresource Technology. 2008;99(17):8394–9. 10.1016/j.biortech.2008.02.039. 10.1016/j.biortech.2008.02.039 18406608

[34] Morgan TJ, Turn SQ, George A. Fast Pyrolysis Behavior of Banagrass as a Function of Temperature and Volatiles Residence Time in a Fluidized Bed Reactor. PLoS ONE. 2015;10(8):e0136511 10.1371/journal.pone.0136511 26308860 PMC4550300

[35] Cui H, Turn SQ, Tran T, Rogers D. Mechanical dewatering and water leaching pretreatment of fresh banagrass, guinea grass, energy cane, and sugar cane: Characterization of fuel properties and byproduct streams. Fuel Processing Technology. 10.1016/j.fuproc.2015.07.027.

[36] Sluiter A HB, Ruiz R, Scarlata J, Templeton D,. Determination of structural carbohydrates and lignin in biomass. Technical Report: National Renewable Energy Laboratory. NREL/TP-510-42618. 2008.

[37] Çetinkol ÖP, Smith-Moritz AM, Cheng G, Lao J, George A, Hong K, et al Structural and Chemical Characterization of Hardwood from Tree Species with Applications as Bioenergy Feedstocks. PLoS ONE. 2012;7(12):e52820 10.1371/journal.pone.0052820 23300786 PMC3532498

[38] Stiles HN, Kandiyoti R. Secondary reactions of flash pyrolysis tars measured in a fluidized-bed pyrolysis reactor with some novel design features. Fuel. 1989;68(Copyright (C) 2012 American Chemical Society (ACS). All Rights Reserved.):275–82. 10.1016/0016-2361(89)90087-2

[39] Tyler RJ. Flash pyrolysis of coals. Fuel. 1979;(58):680.

[40] Mohan D, Pittman CU Jr, Steele PH. Pyrolysis of Wood/Biomass for Bio-oil: A Critical Review. Energy Fuels. 2006;20(Copyright (C) 2012 American Chemical Society (ACS). All Rights Reserved.):848–89. 10.1021/ef0502397

[41] http://www.soest.hawaii.edu/S-LAB/downloads/SLAB_Exeter_PreAcc.pdf. Accessed 7 April 2015.

[42] Eom I-Y, Kim J-Y, Lee S-M, Cho T-S, Yeo H, Choi J-W. Comparison of pyrolytic products produced from inorganic-rich and demineralized rice straw (Oryza sativa L.) by fluidized bed pyrolyzer for future biorefinery approach. Bioresource Technology. 2013;128(0):664–72. 10.1016/j.biortech.2012.09.082. 23220113

[43] Samolada MC, Vasalos IA, editors. Effect of experimental conditions on the composition of gases and liquids from biomass pyrolysis.: Springer; 1993.

[44] Steele PH, Pittman CU Jr, Ingram LL Jr, Gajjela S, Zhang Z, Bhattacharya P, inventors; Mississippi State University, assignee. Method to upgrade bio-oils to fuel and bio-crude. patent US 8,603,199 B2. 2013.

