# Supplementary material for: Fast Pyrolysis of Tropical Biomass Species and Influence of Water Pretreatment on Product Distributions
Source: PLoS One. 2016 Mar 15;11(3):e0151368. doi: 10.1371/journal.pone.0151368 (PMC4792437; doi:10.1371/journal.pone.0151368)
Supplement: S1 File — (DOC) [file pone.0151368.s001.doc]

**Supporting Information - Fast pyrolysis of tropical biomass species and influence of water pretreatment on product distributions**

**S1 File. Temperature distributions across the bed and freeboard**

Table S1.1 to S1.4 present the temperature distributions across the bed (T1 and T2) and the freeboard (T3 to T5) when operating the pyrolysis reactor with different bed positions. The positions of the thermocouples are described in the experimental section of the manuscript. The temperatures displayed in Tables S1.1 to S1.4 are the values noted 5 minutes before feeding the fuel, the temperatures remained within +/- 10 °C of the stated values during the course of an experiment. A three zone furnace is used to heat the reactor with the temperature of each zone set to obtain a uniform as possible temperature across the bed and freeboard. When the reactor is set up for the shortest vapor residence time experiments (bed position BP-4) the bed is located between the top two zones of the furnace which makes it difficult to keep the freeboard at a similar temperature as the bed. To avoid having a dramatically lower temperature in the freeboard than in the bed it was necessary to work with a slightly higher bed temperature than when the bed was in lower positions.

Table A. Temperature distribution across the bed and freeboard for the four different bed positions when operating at 400 °C.

| Thermocouple position | Bed position | | | |
| --- | --- | --- | --- | --- |
|  | BP-1 | BP-2 | BP-3 | BP-4 |
|  | °C | °C | °C | °C |
| T1 | 397 | 409 | 408 | 418 |
| T2 | 401 | 405 | 402 | 406 |
| T3 | 406 | 379 | 410 | 378 |
| T4 | 404 | 422 | 382 | n/a |
| T5 | 404 | 401 | 389 | 354 |
| n/a, not applicable as when the bed is in its highest position (BP-4) thermocouple T4 is no longer in the freeboard of the reactor | | | | |

Table B. Temperature distribution across the bed and freeboard for the four different bed positions when operating at 450 °C.

| Thermocouple position | Bed position | | | |
| --- | --- | --- | --- | --- |
|  | BP-1 | BP-2 | BP-3 | BP-4 |
|  | °C | °C | °C | °C |
| T1 | 451 | 460 | 455 | 471 |
| T2 | 449 | 449 | 453 | 444 |
| T3 | 453 | 408 | 472 | 420 |
| T4 | 449 | 473 | 434 | n/a |
| T5 | 450 | 450 | 440 | 400 |

Table C. Temperature distribution across the bed and freeboard for the four different bed positions when operating at 500 °C.

| Thermocouple position | Bed position | | | |
| --- | --- | --- | --- | --- |
|  | BP-1 | BP-2 | BP-3 | BP-4 |
|  | °C | °C | °C | °C |
| T1 | 500 | 503 | 510 | 520 |
| T2 | 497 | 498 | 506 | 501 |
| T3 | 503 | 459 | 512 | 466 |
| T4 | 498 | 521 | 458 | n/a |
| T5 | 498 | 500 | 475 | 436 |

Table D. Temperature distribution across the bed and freeboard for the four different bed positions when operating at 600 °C.

| Thermocouple position | Bed position | | | |
| --- | --- | --- | --- | --- |
|  | BP-1 | BP-2 | BP-3 | BP-4 |
|  | °C | °C | °C | °C |
| T1 | n/a | 598 | 604 | 614 |
| T2 | n/a | 597 | 600 | 611 |
| T3 | n/a | 554 | 609 | 562 |
| T4 | n/a | 618 | 560 | n/a |
| T5 | n/a | 598 | 567 | 531 |
