# Supplementary material for: Fast Pyrolysis of Tropical Biomass Species and Influence of Water Pretreatment on Product Distributions
Source: PLoS One. 2016 Mar 15;11(3):e0151368. doi: 10.1371/journal.pone.0151368 (PMC4792437; doi:10.1371/journal.pone.0151368)
Supplement: S2 File — (DOC) [file pone.0151368.s002.doc]

**Supporting Information - Fast pyrolysis of tropical biomass species and influence of water pretreatment on product distributions**

**S2 GCMS results**

Tables A to D displays a summary of the quantitative results for the Banagrass pyrolysis oils as a function of vapor residence time (BP-1 to BP-4) and temperature (400, 450, 500 and 600 °C). Table E presents the equivalent data for leucaena, eucalyptus, sugarcane bagasse, energy cane and pretreated energy cane at the shortest residence time (BP-4) at one temperature (450 °C). The values represent the amount of each compound relative to the amount of feedstock (daf) in weight percent. The lower limit of quantification (LLQ) is also given in the tables, which is derived from the calibration data as described in the experimental section of the manuscript. A value of zero means the compound was not detected. Note: the GCMS results presented below are for the absolute yield of each compound present in the bio-oil, which is *not* the same as the 'volatile oil yield' referred to in the manuscript, which is a measure of the amount of material removed from the bio-oils during drying.

In summary of the GCMS results, no single compound in the GC range was found to be present in any of the oil samples in a significant concentration. Instead the chromatograms showed at least ~40 peaks are detected but all at low concentrations. All the bio-oil samples showed a large peak due to 2, 2- dimethoxypropane, which ranged in concentration from ~2 wt% relative to the amount feedstock (daf) at 400 °C, which increased significantly with increasing vapor residence time or increasing temperature, reaching a maximum of ~15 wt% relative to the amount of feedstock (daf) at 600 °C. These results are *not* included in the tables below as it is apparent that 2, 2- dimethoxypropane is a product from reactions between the oil compounds and the solvent (mixture of acetone and methanol). It is possible that some of the 2, 2 dimethoxypropane present in the oils is produced during pyrolysis as other researcher have reported this finding. However, in the present study it was clear that the concentration of 2, 2 dimethoxypropane greatly increased in the samples over time, this could be clearly seen from the three analyses that are performed on each sample, with the first analysis always containing a lower concentration than subsequent analyses.

A brief aging study was performed and the concentration of 2, 2 dimethoxypropane in the oil solutions keep increasing over a period of at least 10 days, with the biggest increases observed within the first few hours of recovering the bio-oils with smaller increases over extended lengths of time. Comparing the GCMS results for the untreated and pretreated banagrass shows that the amount of 2, 2 dimethoxypropane is significantly lower in the bio-oils from the pretreated banagrass (2 to 15 wt%) than untreated banagrass (5 to 80 wt%). This is an indication that the bio-oil from pretreated banagrass is more stable (less aging reactions) than the bio-oil from untreated banagrass.

Table A. Quantitative GCMS results for the pretreated banagrass bio-oils recovered at the longest residence time (BP-1) over four temperatures. Results are presented as wt% relative to the amount of feedstock (daf).

| Target Compounds | *LLQ* | Temperature / °C | | |
| --- | --- | --- | --- | --- |
|  |  | 500 | 450 | 400 |
|  | *ug/mL* | wt% | wt% | wt% |
| Cyclohexane | *5* | 0 | 0 | 0 |
| Furfural | *15* | <LLQ | <LLQ | <LLQ |
| 3-methyl-2-cyclopenten-1-one | *15* | 0 | <LLQ | 0 |
| Phenol | *5* | 0 | <LLQ | <LLQ |
| 2-methoxy-phenol | *10* | <LLQ | <LLQ | <LLQ |
| o-Cresol | *10* | <LLQ | <LLQ | <LLQ |
| p-Cresol | *10* | <LLQ | <LLQ | <LLQ |
| m-Cresol | *5* | <LLQ | <LLQ | <LLQ |
| Creosol | *5* | <LLQ | <LLQ | <LLQ |
| 2,4-Dimethyl-phenol | *5* | <LLQ | <LLQ | 0 |
| 4-ethyl-phenol, | *10* | <LLQ | <LLQ | <LLQ |
| 2,6-dimethoxy-phenol, | *5* | <LLQ | 0.07 | 0.08 |
| Indole | *5* | <LLQ | <LLQ | <LLQ |
| Isoeugenol | *10* | <LLQ | <LLQ | <LLQ |
| Benzene | *10* | 0 | <LLQ | 0 |
| Naphthalene | *10* | <LLQ | <LLQ | <LLQ |
| Repeatability is +/- 0.3 wt% of the absolute values.  <LLQ, less than the lower limit of quantification.  0 means the compound was not detected. | | | | |

Table B. Quantitative GCMS results for the pretreated banagrass bio-oils recovered at the second longest residence time (BP-2) over four temperatures. Results are presented as wt% relative to the amount of feedstock (daf).

| Target Compounds | LLQ | Temperature / °C | | | |
| --- | --- | --- | --- | --- | --- |
|  |  | 600 | 500 | 450 | 400 |
|  | ug/mL | wt% | wt% | wt% | wt% |
| Cyclohexane | 5 | 0 | 0 | 0 | 0 |
| Furfural | 15 | <LLQ | <LLQ | <LLQ | <LLQ |
| 3-methyl-2-cyclopenten-1-one | 15 | <LLQ | <LLQ | <LLQ | <LLQ |
| Phenol | 5 | 0.15 | 0.24 | 0.11 | 0 |
| 2-methoxy-phenol | 10 | <LLQ | 0.16 | 0.18 | 0.19 |
| o-Cresol | 10 | <LLQ | <LLQ | <LLQ | <LLQ |
| p-Cresol | 10 | <LLQ | <LLQ | <LLQ | <LLQ |
| m-Cresol | 5 | 0.07 | <LLQ | 0.07 | 0.08 |
| Creosol | 5 | 0.09 | 0.12 | 0.18 | 0.18 |
| 2,4-Dimethyl-phenol | 5 | <LLQ | 0.11 | 0.17 | 0.15 |
| 4-ethyl-phenol, | 10 | 0.16 | 0.19 | 0.17 | 0.18 |
| 2,6-dimethoxy-phenol, | 5 | 0.18 | 0.18 | 0.23 | 0.22 |
| Indole | 5 | <LLQ | <LLQ | 0.11 | <LLQ |
| Isoeugenol | 10 | 0.36 | 0.31 | 0.35 | 0.37 |
| Benzene | 10 | <LLQ | <LLQ | 0 | <LLQ |
| Naphthalene | 10 | <LLQ | <LLQ | <LLQ | <LLQ |
| Repeatability is +/- 0.3 wt% of the absolute values.  <LLQ, less than the lower limit of quantification.  0 means the compound was not detected. | | | | | |

Table C. Quantitative GCMS results for the pretreated banagrass bio-oils recovered at the second shortest residence time (BP-3) over four temperatures. Results are presented as wt% relative to the amount of feedstock (daf).

| **Target Compounds** | LLQ | Temperature / °C | | | |
| --- | --- | --- | --- | --- | --- |
|  |  | 600 | 500 | 450 | 400 |
|  | ug/mL | wt% | wt% | wt% | wt% |
| Cyclohexane | 5 | 0 | 0 | 0 | 0 |
| Furfural | 15 | 0 | <LLQ | <LLQ | <LLQ |
| 3-methyl-2-cyclopenten-1-one | 15 | <LLQ | <LLQ | 0 | <LLQ |
| Phenol | 5 | 0 | <LLQ | 0.10 | 0.19 |
| 2-methoxy-phenol | 10 | <LLQ | <LLQ | 0.17 | 0.16 |
| o-Cresol | 10 | <LLQ | <LLQ | <LLQ | <LLQ |
| p-Cresol | 10 | <LLQ | 0.15 | 0.14 | 0.15 |
| m-Cresol | 5 | 0.08 | <LLQ | 0.07 | 0.09 |
| Creosol | 5 | <LLQ | 0.08 | 0.16 | 0.19 |
| 2,4-Dimethyl-phenol | 5 | 0.07 | <LLQ | 0.10 | <LLQ |
| 4-ethyl-phenol, | 10 | 0.19 | 0.17 | 0.18 | 0.19 |
| 2,6-dimethoxy-phenol, | 5 | <LLQ | 0.17 | 0.23 | 0.25 |
| Indole | 5 | <LLQ | 0 | 0.08 | <LLQ |
| Isoeugenol | 10 | 0.14 | 0.19 | 0.35 | 0.38 |
| Benzene | 10 | <LLQ | <LLQ | 0 | 0 |
| Naphthalene | 10 | <LLQ | <LLQ | <LLQ | <LLQ |
| Repeatability is +/- 0.3 wt% of the absolute values.  <LLQ, less than the lower limit of quantification.  0 means the compound was not detected. | | | | | |

Table D. Quantitative GCMS results for the pretreated banagrass bio-oils recovered at the shortest residence time (BP-4) over four temperatures. Results are presented as wt% relative to the amount of feedstock (daf).

| Target Compounds | LLQ | Temperature / °C | | | |
| --- | --- | --- | --- | --- | --- |
|  |  | 600 | 500 | 450 | 400 |
|  | ug/mL | wt% | wt% | wt% | wt% |
| Cyclohexane | 5 | 0 | 0 | 0 | 0 |
| Furfural | 15 | <LLQ | <LLQ | <LLQ | 0 |
| 3-methyl-2-cyclopenten-1-one | 15 | <LLQ | <LLQ | <LLQ | 0 |
| Phenol | 5 | 0.20 | 0 | 0.10 | <LLQ |
| 2-methoxy-phenol | 10 | <LLQ | 0.18 | <LLQ | <LLQ |
| o-Cresol | 10 | <LLQ | <LLQ | <LLQ | <LLQ |
| p-Cresol | 10 | <LLQ | <LLQ | <LLQ | <LLQ |
| m-Cresol | 5 | 0.09 | 0.11 | 0.12 | <LLQ |
| Creosol | 5 | 0 | 0.12 | <LLQ | <LLQ |
| 2,4-Dimethyl-phenol | 5 | 0.09 | 0.15 | <LLQ | <LLQ |
| 4-ethyl-phenol, | 10 | 0.20 | 0.23 | 0.28 | 0.24 |
| 2,6-dimethoxy-phenol, | 5 | <LLQ | 0.22 | <LLQ | 0.14 |
| Indole | 5 | 0 | <LLQ | <LLQ | 0 |
| Isoeugenol | 10 | <LLQ | 0.35 | <LLQ | <LLQ |
| Benzene | 10 | 0 | 0 | 0 | 0 |
| Naphthalene | 10 | <LLQ | 0 | <LLQ | 0 |
| Repeatability is +/- 0.3 wt% of the absolute values.  <LLQ, less than the lower limit of quantification.  0 means the compound was not detected. | | | | | |

Table E. Quantitative GCMS results for leucaena, eucalyptus, sugarcane bagasse, energy cane and pretreated energy cane (S3) bio-oils recovered at the shortest residence time (BP-4) at 450 °C. Results are presented as wt% relative to the amount of feedstock (daf).

| Target Compounds | LLQ | Leucaena | Eucalyptus | S-Bagasse | E-cane | E-Cane S3 |
| --- | --- | --- | --- | --- | --- | --- |
|  | ug/mL | wt% | wt% | wt% | wt% | wt% |
| Cyclohexane | 5 | 0 | 0 | 0 | 0 | 0 |
| Furfural | 15 | 0 | <LLQ | 0 | <LLQ | 0 |
| 3-methyl-2-cyclopenten-1-one | 15 | <LLQ | 0 | 0 | 0 | <LLQ |
| Phenol | 5 | 0 | 0 | 0.08 | 0.09 | 0 |
| 2-methoxy-phenol | 10 | <LLQ | <LLQ | 0.15 | <LLQ | 0.11 |
| o-Cresol | 10 | <LLQ | <LLQ | <LLQ | <LLQ | <LLQ |
| p-Cresol | 10 | <LLQ | 0 | <LLQ | 0 | 0 |
| m-Cresol | 5 | 0 | 0 | <LLQ | 0 | 0 |
| Creosol | 5 | 0.15 | 0.15 | 0.13 | <LLQ | 0.14 |
| 2,4-Dimethyl-phenol | 5 | 0.12 | 0.13 | <LLQ | 0.09 | <LLQ |
| 4-ethyl-phenol, | 10 | 0.18 | <LLQ | 0.20 | 0.17 | 0.15 |
| 2,6-dimethoxy-phenol, | 5 | 0.21 | 0.19 | 0.20 | 0.21 | 0.20 |
| Indole | 5 | <LLQ | <LLQ | <LLQ | <LLQ | 0.07 |
| Isoeugenol | 10 | 0.30 | 0.29 | 0.31 | 0.31 | 0.28 |
| Benzene | 10 | 0 | <LLQ | <LLQ | <LLQ | <LLQ |
| Naphthalene | 10 | <LLQ | <LLQ | <LLQ | <LLQ | <LLQ |
| Repeatability is +/- 0.3 wt% of the absolute values.  <LLQ, less than the lower limit of quantification.  0 means the compound was not detected. | | | | | | |

1. Steele PH, Pittman CU, Jr., Ingram LL, Jr., Gajjela S, Zhang Z, Bhattacharya P, inventors; Mississippi State University, assignee. Method to upgrade bio-oils to fuel and bio-crude. patent US 8,603,199 B2. 2013.

2. Morgan TJ, Turn SQ, George A. Fast Pyrolysis Behavior of Banagrass as a Function of Temperature and Volatiles Residence Time in a Fluidized Bed Reactor. PLoS ONE. 2015;10(8):e0136511. doi: 10.1371/journal.pone.0136511.
