# Supplementary material for: Fast Pyrolysis of Tropical Biomass Species and Influence of Water Pretreatment on Product Distributions
Source: PLoS One. 2016 Mar 15;11(3):e0151368. doi: 10.1371/journal.pone.0151368 (PMC4792437; doi:10.1371/journal.pone.0151368)
Supplement: S3 File — (DOC) [file pone.0151368.s003.doc]

**Supporting Information - Fast pyrolysis of tropical biomass species and influence of water pretreatment on product distributions**

**S3 Elemental analysis of the biomass ashes - Tabulated**

Table A. Elemental analysis of the ash from leucaena, eucalyptus, sugarcane bagasse, energy cane and pretreated energy cane (S3), banagrass and pretreated banagrass (S3), the ash was calcined at 600 °C prior to analysis. Presented as wt% of the ash.

| Sample | Element | SiO2 | Al2O3 | TiO2 | Fe2O3 | CaO | MgO | Na2O | K2O | P2O5 | SO3 | Cl | CO2 | *Sum* |
| --- | --- | --- | --- | --- | --- | --- | --- | --- | --- | --- | --- | --- | --- | --- |
| Leucaena | wt% | 16.7 | 5.7 | 0.1 | 7.4 | 24.2 | 9.3 | 1.7 | 17.3 | 4.0 | 0.8 | 3.6 | 6.6 | *90.7* |
| Eucalyptus | wt% | 2.8 | 2.9 | 0.1 | 5.6 | 31.3 | 5.7 | 6.1 | 12.4 | 11.4 | 1.7 | 2.1 | 15.2 | *82.2* |
| S-Bagasse | wt% | 39.4 | 21.6 | 3.5 | 19.5 | 2.4 | 1.3 | 0.7 | 2.1 | 1.4 | 0.7 | <0.01 | 0.3 | *92.7* |
| E-Cane | wt% | 62.2 | 0.8 | 0.02 | 0.5 | 6.2 | 1.6 | 1.7 | 14.4 | 3.6 | 7.8 | 2.7 | 0.4 | *101.4* |
| E-Cane S3 | wt% | 67.8 | 1.2 | 0.1 | 1.9 | 3.0 | 1.0 | 0.7 | 4.3 | 1.5 | 1.6 | 0.0 | 0.4 | *83.1* |
| Banagrass | wt% | 47.1 | 0.8 | <0.01 | 0.5 | 2.6 | 2.5 | 0.5 | 27.0 | 6.1 | 1.5 | 12.1 | 0.8 | *100.7* |
| Banagrass S3 | wt% | 61.6 | 1.4 | <0.01 | 1.7 | 4.1 | 1.3 | 0.6 | 6.5 | 2.5 | 0.7 | 0.3 | 1.0 | *80.7* |
| Repeatability is estimated to be less than +/- 0.5 % of the absolute value,  It is not clear why some of the ashes only sum to ~80 wt%, possible due to an underestimation of silica. | | | | | | | | | | | | | | |
