# Supplementary material for: Fast Pyrolysis of Tropical Biomass Species and Influence of Water Pretreatment on Product Distributions
Source: PLoS One. 2016 Mar 15;11(3):e0151368. doi: 10.1371/journal.pone.0151368 (PMC4792437; doi:10.1371/journal.pone.0151368)
Supplement: S4 File — (DOC) [file pone.0151368.s004.doc]

**Supporting Information - Fast pyrolysis of tropical biomass species and influence of water pretreatment on product distributions**

**S4 Fast pyrolysis product yields (bio-oil, char and gas) - Tabulated**

Table A. Bio-oil, char and gas yields (wt% feedstock daf) from pyrolysis of pretreated banagrass at the longest residence time (BP-1)

| Temperature | BP | Dry  bio-oil^¥^ | Volatile  bio-oil^#^ | Char_org_* | ^CO CO_2_ CH_4_ H_2_ | Undetected** |
| --- | --- | --- | --- | --- | --- | --- |
| C | Second | wt% | wt% | wt% | wt% | wt% |
| 400 | BP-1 | 33.3 | <LLQ | 7.2 | 9.1 | 50.4 |
| 450 | BP-1 | 35.2 | <LLQ | 4.6 | 12.3 | 47.9 |
| 500 | BP-1 | 24.4 | <LLQ | 4.9 | 19.0 | 51.7 |
| ^¥^ S.D. of the 'dry bio-oil' yield is < ±2.0 wt% (absolute).  ^#^ Volatile bio-oil refers to the amount of bio-oil removed from the sample during drying and is determined by analyzing the bio-oil solution by GCMS before drying and again after it is dried.  <LLQ, less than the lower limit of quantification, which equates to a yield of less than 2.0 wt% of the 'daf' feedstock.  ^ Indicative values derived from on-line gas analysis.  * The bias in the char yield is estimated to be < ±2 % (absolute) and S.D. < ±1.5 wt%.  ** 'Undetected' is derived as: 100% - (Dry bio-oil + Volatile bio-oil + Char + CO, CO_2_, CH_4_ and H_2_ yields). | | | | | | |

Table B. Bio-oil, char and gas yields (wt% feedstock daf) from pyrolysis of pretreated banagrass at the 2nd longest residence time (BP-2)

| Temperature | BP | Dry  bio-oil^¥^ | Volatile  bio-oil^#^ | Char_org_* | ^CO CO_2_ CH_4_ H_2_ | Undetected** |
| --- | --- | --- | --- | --- | --- | --- |
| C | Second | wt% | wt% | wt% | wt% | wt% |
| 400 | BP-2 | 36.2 | <LLQ | 7.5 | 6.7 | 49.6 |
| 450 | BP-2 | 36.4 | <LLQ | 5.6 | 8.6 | 49.4 |
| 500 | BP-2 | 29.4 | 0.1 | 3.8 | 12.7 | 53.9 |
| 600 | BP-2 | 14.1 | <LLQ | 0.8 | 29.0 | 56.0 |
| ^¥^ S.D. of the 'dry bio-oil' yield is < ±2.0 wt% (absolute).  ^#^ Volatile bio-oil refers to the amount of bio-oil removed from the sample during drying and is determined by analyzing the bio-oil solution by GCMS before drying and again after it is dried.  <LLQ, less than the lower limit of quantification, which equates to a yield of less than 2.0 wt% of the 'daf' feedstock.  ^ Indicative values derived from on-line gas analysis.  * The bias in the char yield is estimated to be < ±2 % (absolute) and S.D. < ±1.5 wt%.  ** 'Undetected' is derived as: 100% - (Dry bio-oil + Volatile bio-oil + Char + CO, CO_2_, CH_4_ and H_2_ yields). | | | | | | |

Table C. Bio-oil, char and gas yields (wt% feedstock daf) from pyrolysis of pretreated banagrass at the 2nd shortest residence time (BP-3)

| Temperature | BP | Dry  bio-oil^¥^ | Volatile  bio-oil^#^ | Char_org_* | ^CO CO_2_ CH_4_ H_2_ | Undetected** |
| --- | --- | --- | --- | --- | --- | --- |
| C | Second | wt% | wt% | wt% | wt% | wt% |
| 400 | BP-3 | 35.6 | 0.1 | 6.6 | 7.1 | 50.5 |
| 450 | BP-3 | 36.2 | <LLQ | 5.5 | 9.9 | 48.5 |
| 500 | BP-3 | 32.8 | <LLQ | 4.6 | 12.6 | 50.0 |
| 600 | BP-3 | 15.5 | <LLQ | 1.8 | 28.6 | 54.2 |
| ^¥^ S.D. of the 'dry bio-oil' yield is < ±2.0 wt% (absolute).  ^#^ Volatile bio-oil refers to the amount of bio-oil removed from the sample during drying and is determined by analyzing the bio-oil solution by GCMS before drying and again after it is dried.  <LLQ, less than the lower limit of quantification, which equates to a yield of less than 2.0 wt% of the 'daf' feedstock.  ^ Indicative values derived from on-line gas analysis.  * The bias in the char yield is estimated to be < ±2 % (absolute) and S.D. < ±1.5 wt%.  ** 'Undetected' is derived as: 100% - (Dry bio-oil + Volatile bio-oil + Char + CO, CO_2_, CH_4_ and H_2_ yields). | | | | | | |

Table D. Bio-oil, char and gas yields (wt% feedstock daf) from pyrolysis of pretreated banagrass at the shortest residence time (BP-4)

| Temperature | BP | Dry  bio-oil^¥^ | Volatile  bio-oil^#^ | Char_org_* | ^CO CO_2_ CH_4_ H_2_ | Undetected** |
| --- | --- | --- | --- | --- | --- | --- |
| C | Second | wt% | wt% | wt% | wt% | wt% |
| 400 | BP-4 | 38.7 | 0.4 | 4.1 | 5.1 | 51.7 |
| 450 | BP-4 | 41.3 | <LLQ | 3.5 | 7.4 | 47.8 |
| 500 | BP-4 | 37.3 | 0.8 | - | 9.5 | - |
| 600 | BP-4 | 28.3 | 0.2 | 1.9 | 16.2 | 53.5 |
| ^¥^ S.D. of the 'dry bio-oil' yield is < ±2.0 wt% (absolute).  ^#^ Volatile bio-oil refers to the amount of bio-oil removed from the sample during drying and is determined by analyzing the bio-oil solution by GCMS before drying and again after it is dried.  <LLQ, less than the lower limit of quantification, which equates to a yield of less than 2.0 wt% of the 'daf' feedstock.  ^ Indicative values derived from on-line gas analysis.  * The bias in the char yield is estimated to be < ±2 % (absolute) and S.D. < ±1.5 wt%.  ** 'Undetected' is derived as: 100% - (Dry bio-oil + Volatile bio-oil + Char + CO, CO_2_, CH_4_ and H_2_ yields). | | | | | | |
