# Supplementary material for: Fast Pyrolysis of Tropical Biomass Species and Influence of Water Pretreatment on Product Distributions
Source: PLoS One. 2016 Mar 15;11(3):e0151368. doi: 10.1371/journal.pone.0151368 (PMC4792437; doi:10.1371/journal.pone.0151368)
Supplement: S5 File — (DOC) [file pone.0151368.s005.doc]

**Supporting Information - Fast pyrolysis of tropical biomass species and influence of water pretreatment on product distributions**

**S5 Permanent gas - data tables**

Table A. Permanent gas data from the pyrolysis of pretreated banagrass at the longest residence time (BP-1). Data presented as wt% relative to the daf feedstock.

| Temp | CO | CO2 | CH4 | H2 | Total Producer Gas |
| --- | --- | --- | --- | --- | --- |
| C | wt% | wt% | wt% | wt% | L/g daf |
| 400 | 6.0 | 2.8 | 0.3 | 0.02 | 0.08 |
| 450 | 8.8 | 2.8 | 0.6 | 0.07 | 0.11 |
| 500 | 14.8 | 3.0 | 1.0 | 0.20 | 0.19 |

Table B. Permanent gas data from the pyrolysis of pretreated banagrass at the second longest residence time (BP-2). Data presented as wt% relative to the daf feedstock.

| Temp | CO | CO2 | CH4 | H2 | Total Producer Gas |
| --- | --- | --- | --- | --- | --- |
| C | wt% | wt% | wt% | wt% | L/g daf |
| 400 | 2.9 | 1.6 | 0.1 | 0.01 | 0.04 |
| 450 | 6.1 | 2.1 | 0.4 | 0.03 | 0.08 |
| 500 | 9.5 | 2.4 | 0.7 | 0.12 | 0.12 |
| 600 | 24.4 | 3.6 | 1.3 | 0.49 | 0.31 |

Table C. Permanent gas data from the pyrolysis of pretreated banagrass at the second shortest residence time (BP-3). Data presented as wt% relative to the daf feedstock.

| Temp | CO | CO2 | CH4 | H2 | Total Producer Gas |
| --- | --- | --- | --- | --- | --- |
| C | wt% | wt% | wt% | wt% | L/g daf |
| 400 | 4.6 | 2.1 | 0.2 | 0.02 | 0.06 |
| 450 | 6.9 | 2.1 | 0.5 | 0.05 | 0.09 |
| 500 | 9.0 | 2.4 | 0.6 | 0.08 | 0.11 |
| 600 | 20.3 | 3.0 | 1.5 | 0.33 | 0.27 |

Table D. Permanent gas data from the pyrolysis of pretreated banagrass at the shortest residence time (BP-4). Data presented as wt% relative to the daf feedstock.

| Temp | CO | CO2 | CH4 | H2 | Total Producer Gas |
| --- | --- | --- | --- | --- | --- |
| C | wt% | wt% | wt% | wt% | L/g daf |
| 400 | 3.6 | 1.3 | 0.2 | 0.01 | 0.04 |
| 450 | 4.8 | 2.3 | 0.3 | 0.02 | 0.06 |
| 500 | 6.8 | 2.1 | 0.5 | 0.04 | 0.08 |
| 600 | 12.8 | 2.4 | 0.8 | 0.14 | 0.16 |
